# A comprehensive assessment of inbreeding and laboratory adaptation in *Aedes aegypti* mosquitoes

**DOI:** 10.1111/eva.12740

**Published:** 2018-12-17

**Authors:** Perran A. Ross, Nancy M. Endersby‐Harshman, Ary A. Hoffmann

**Affiliations:** ^1^ Bio21 Institute and the School of BioSciences The University of Melbourne Parkville Victoria Australia

**Keywords:** *Aedes aegypti*, biological control, colonization, inbreeding, laboratory adaptation

## Abstract

Modified *Aedes aegypti* mosquitoes reared in laboratories are being released around the world to control wild mosquito populations and the diseases they transmit. Several efforts have failed due to poor competitiveness of the released mosquitoes. We hypothesized that colonized mosquito populations could suffer from inbreeding depression and adapt to laboratory conditions, reducing their performance in the field. We established replicate populations of *Ae. aegypti *mosquitoes collected from Queensland, Australia, and maintained them in the laboratory for twelve generations at different census sizes. Mosquito colonies maintained at small census sizes (≤100 individuals) suffered from inbreeding depression due to low effective population sizes which were only 25% of the census size as estimated by SNP markers. Populations that underwent full‐sib mating for nine consecutive generations had greatly reduced performance across all traits measured. We compared the established laboratory populations with their ancestral population resurrected from quiescent eggs for evidence of laboratory adaptation. The overall performance of laboratory populations maintained at a large census size (400 individuals) increased, potentially reflecting adaptation to artificial rearing conditions. However, most individual traits were unaffected, and patterns of adaptation were not consistent across populations. Differences between replicate populations may indicate that founder effects and drift affect experimental outcomes. Though we find limited evidence of laboratory adaptation, mosquitoes maintained at low population sizes can clearly suffer fitness costs, compromising the success of “rear‐and‐release” strategies for arbovirus control.

## 1 INTRODUCTION

*Aedes aegypti* mosquitoes transmit some of the most important arboviruses in the world, including dengue, Zika, and chikungunya. These diseases are an enormous burden to global health, and the eradication or disruption of their vectors is currently the leading approach to their control. Several of these strategies rely on rearing and releasing modified mosquitoes into the environment to reduce disease incidence. The sterile insect technique has been used for decades to suppress mosquito populations, though many programs using this technique have not succeeded in achieving substantial population suppression (Bellini et al., 2007; Bellini, Medici, Puggioli, Balestrino, & Carrieri, 2013; Benedict & Robinson, 2003). In this approach, male mosquitoes are irradiated or chemically treated and then released into the field in large numbers to sterilize the wild females. Alternatives to this technique have recently emerged which do not rely on traditional sterilization (reviewed in McGraw & O'Neill, 2013; Ritchie & Johnson, 2017). Transgenic *Ae. aegypti* males possessing a dominant lethal system have been released in multiple locations where they have reduced population sizes, at least in the short term (Carvalho et al., 2015; Garziera et al., 2017; Harris et al., 2012; Lacroix et al., 2012). When these males mate with wild females, most offspring die before reaching the late pupal stage, though a low proportion can emerge as functional adults (Curtis et al., 2015) and may persist for months after releases cease (Garziera et al., 2017). *Aedes* mosquitoes infected experimentally with the intracellular bacterium *Wolbachia *are also being released into the field for disease control programs. Certain strains of *Wolbachia* reduce the capacity for mosquitoes to transmit RNA viruses (Ferguson et al., 2015), and infected males can effectively sterilize wild, uninfected females through cytoplasmic incompatibility (Walker et al., 2011; Xi, Khoo, & Dobson, 2005). Mosquitoes infected with *Wolbachia* are now being released into the field, both to suppress populations (Mains, Brelsfoard, Rose, & Dobson, 2016; O'Connor et al., 2012) and to replace populations with mosquitoes that are refractory to virus transmission (Hoffmann et al., 2011; Nguyen et al., 2015; Schmidt, et al., 2017).

Rear‐and‐release approaches to arbovirus control require large quantities of mosquitoes to be reared in the laboratory for eventual release into the field. For sterile and incompatible male approaches, high ratios of modified to wild males are needed to achieve suppression, particularly if the modifications have deleterious effects on male fitness (Winskill et al., 2014). Laboratory environments are inherently artificial, and colonized mosquito populations experience an entirely different set of selective pressures compared to natural populations (Leftwich, Bolton, & Chapman, 2016). Many laboratory mosquito populations are held at a controlled temperature, humidity, and photoperiod, provided with abundant nutrition, and reared in discrete generations according to a schedule (Benedict, 1997; Carvalho et al., 2014; Munstermann, 1997). Rearing insects in discrete generations may select for an earlier, shorter, and more productive reproductive period, as only individuals that adhere to the rearing schedule will contribute to the next generation (Sgro & Partridge, 2000; Simoes, Santos, & Matos, 2009). Laboratory populations of insects are often maintained at high adult densities due to space limitation which could lead to intense male–male competition and altered courtship behavior (Pereira et al., 2007; Reisen, Knop, & Peloquin, 1985; Rull, Brunel, & Mendez, 2005). Laboratory environments can also lack selective pressures which could lead to declines in later life reproduction (Bryant & Reed, 1999), a reduced ability to survive temperature extremes, dry conditions, or starvation (Hoffmann, Hallas, Sinclair, & Partridge, 2001), or a loss of insecticide resistance (Grossmann et al., 2018; Pimentel, Schwardt, & Dewey, 1953). Maintaining populations in the laboratory can also cause a reduction in genetic diversity resulting in low adaptive potential and inbreeding depression (Briscoe et al., 1992). Laboratory environments can therefore impose rapid genetic changes on insect populations, and laboratory‐derived mosquitoes could be mal‐adapted to the target population when eventually released into the field (Frankham, 2008).

Competitive mosquitoes are critical for the success of rear‐and‐release programs. Past sterile insect interventions have failed due to the poor performance of released mosquitoes, possibly caused by laboratory adaptation (Benedict & Robinson, 2003; Helinski & Harrington, 2013; Reisen et al., 1982). For releases of sterile, dominant lethal, or incompatible males, the ability of modified males to seek and inseminate wild females is especially important (Chambers, Hapairai, Peel, Bossin, & Dobson, 2011; Harris et al., 2011). For approaches where modified mosquitoes are intended to persist in the environment, it is often necessary for them to perform similarly to wild mosquitoes. Two attempts to establish the *w*MelPop *Wolbachia* infection in natural *Ae. aegypti* populations failed due to deleterious effects associated with the infection, including costs to fecundity, adult lifespan, and egg viability (Nguyen et al., 2015). While trait variation related to fitness in mosquitoes has often been well‐characterized, there are fewer attempts to compare laboratory strains intended for release against the wild mosquitoes against which they are intended to compete.

Across all mosquito species, there are numerous studies that compare life history, morphological, and physiological traits between laboratory and field populations for evidence of laboratory adaptation (Supporting Information Table S1). Substantial and rapid adaptation by mosquitoes to laboratory conditions is often observed (e.g., Watson, Marshall, & Kay, 2000; Oliva, Benedict, Lempérière, & Gilles, 2011), but there are several instances of laboratory populations suffering reduced fitness (e.g., Huho et al., 2007; Ponlawat & Harrington, 2007). Other studies find no clear differences between laboratory and field populations despite years of separation (e.g., Hassan, El‐Motasim, Ahmed, & El‐Sayed, 2010; Faull & Williams, 2015; Jong, Kassim, Naziri, & Webb, 2017). Mosquitoes maintained in the laboratory can differ from wild populations for many traits, including blood‐feeding duration (Chadee & Beier, 1997; Chadee, Beier, & Mohammed, 2002), wing shape (Yeap, Endersby, Johnson, Ritchie, & Hoffmann, 2013), oviposition behavior (Allgood & Yee, 2017), mating success (Haeger & O'Meara, 1970; Knop, Asman, Reisen, & Milby, 1987; Lima, Valle, & Peixoto, 2004), swarming behavior (Reisen et al., 1985), and susceptibility to pathogens (Grimstad, Craig, Ross, & Yuill, 1977; Lorenz, Beaty, Aitken, Wallis, & Tabachnick, 1984; Salazar, Richardson, Sanchez‐Vargas, Olson, & Beaty, 2007; Vazeille, Rosen, Mousson, & Failloux, 2003). Researchers often compare a single wild population to a single long‐established laboratory population (e.g., Haeger & O'Meara, 1970; Lima et al., 2004), but these results could be confounded by inbreeding, drift, and bottlenecks in the laboratory population rather than reflecting laboratory adaptation. Differences between populations could also be affected by rearing conditions, for example, if the wild population is reared under field conditions (e.g., Huho et al., 2007; Oliva et al., 2011; Ng'habi et al., 2015) or if measurements are conducted at different time points (e.g., Lorenz et al., 1984; Chadee et al., 2002). Other studies compare populations collected from different locations (e.g., Salazar et al., 2007; Allgood & Yee, 2014; Allgood & Yee, 2017), and any effects of laboratory maintenance could be confounded by local adaptation.

The extent of laboratory adaptation can vary between insect orders (Hoffmann & Ross, 2018), and this could reflect differences in the range of conditions that can be tolerated relative to the conditions experienced in the laboratory (Ochieng'‐Odero, 1994). Laboratory environments that are suboptimal will impose strong selective pressures on mosquito populations, leading to rapid adaptation (e.g., Watson et al., 2000). Colonized mosquito species can require a specific set of conditions such as swarm markers (Watson et al., 2000), artificial horizons (Marchand, 1985), dusk periods (Marchand, 1985), or exposure to stroboscopic light (Lardeux et al., 2007) to improve their reproductive success in the laboratory. Other species will not freely reproduce in the laboratory at all, requiring induced copulation over successive generations before free‐mating colonies can be established (Bryan & Southgate, 1978; McDaniel & Horsfall, 1957). In contrast, *Ae. aegypti* collected from the field perform well in the laboratory without any of these specific requirements (e.g., Munstermann, 1997), and therefore, less drastic differences in traits would be expected between laboratory and field populations due to a lack of selective pressures.

Rear‐and‐release programs with modified *Ae. aegypti* mosquitoes are now underway in several countries, and many of these programs rely on the use of mosquitoes that have been inbred or maintained in the laboratory for extended periods. We colonized replicate *Ae. aegypti* populations collected from Queensland, Australia, to assess the effects of laboratory maintenance and inbreeding on life history traits in this species. We find that inbreeding is costly and is associated with a reduction in effective population size, but we find limited evidence of laboratory adaptation for most life history traits. Modified mosquitoes reared for disease control programs should therefore be maintained at large population sizes and/or crossed with field populations prior to field release. Our research highlights potential issues with maintaining colonized insects that are destined for field release, and informs protocols for the maintenance of *Ae. aegypti *in the laboratory.

## 2 MATERIALS AND METHODS

### 2.1 Replicate population establishment

**Figure 1:**
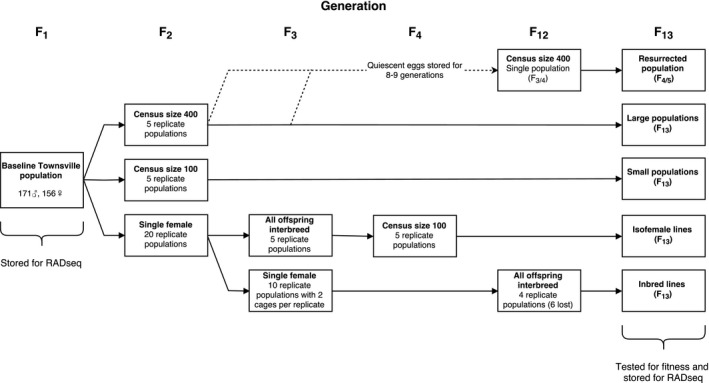
Maintenance scheme for replicate *Aedes aegypti *laboratory populations. An ancestral population was established from eggs collected from Townsville, Australia, that all other populations were derived from. Replicate populations were maintained separately beginning from F_2_ and were not interbred

*Aedes *eggs collected from ovitraps near Townsville, Australia, in September 2015 (Ritchie, 2001) were hatched and reared in the laboratory (see *Colony maintenance*). *Aedes aegypti *larvae were separated from other species based on an identification key (Rueda, 2004). A total of 327 *Ae. aegypti *adults (171 males and 156 females) were obtained and added to a single 19.7‐L (27 cm^3^) BugDorm‐1^®^ colony cage (MegaView Science Co., Ltd., Taichung City, Taiwan). Females were blood‐fed, and all eggs laid were pooled and hatched in a single plastic tray (30 × 20 × 10 cm) containing 3 L of water. Larvae were selected at random and divided into groups to establish replicate populations (Figure 1). Five populations were maintained at a census size of 400 adults (large populations), and five populations were maintained at a census size of 100 adults (small populations). Twenty adult females were also isolated for oviposition. The offspring from five isolated females were used to establish five additional populations maintained at a census size of 100 adults (isofemale lines), while the offspring from 10 females were maintained with a single male and female each (inbred lines). At least two mating pairs were established for each inbred line (Supporting Information Table S2), but only a single pair was used to found the next generation. Their offspring underwent full‐sib mating each generation for nine generations, and then all progeny were interbred during F_12_ to build up numbers for experiments. All replicate populations were maintained until F_13_ when experimental comparisons were performed. All adults from the ancestral Townsville population (F_1_) and the replicate populations at F_13_ were stored in absolute ethanol at −20°C for pooled double‐digest RADseq. Only two inbred lines had sufficient numbers for RADseq due to the loss of most inbred lines over the course of full‐sib mating (Supporting Information Table S2).

*Aedes aegypti *eggs can withstand desiccation and remain viable for up to 1 year (Faull & Williams, 2015). We utilized this ability to perform direct comparisons between the ancestral population and the derived populations simultaneously. Eggs laid by F_2_ and F_3_ females were stored under humid conditions for 7–8 months at 26°C and then hatched at the same time as eggs laid by F_11_ females from the other populations. A colony derived from larvae that hatched was maintained under standard conditions for one generation, and their progeny (F_4/5_) were used for experiments alongside the populations at F_13_. We used a relaxed generation to avoid deleterious effects associated with extended quiescence (Perez & Noriega, 2012); however, we cannot rule out any indirect effects on fitness. Colonies derived from eggs collected from Cairns and Innisfail, Australia, were also used for experimental comparisons. These colonies were maintained as single caged populations with a census size of 400 individuals. Quiescent eggs from the Innisfail population were also used to generate a colony that had experienced fewer generations of maintenance under laboratory conditions. Eggs collected from Cairns at a later stage were used to establish a colony for comparisons with the Cairns colony at F_22_.

### 2.2 Colony maintenance

All populations were maintained in a controlled temperature laboratory environment (26 ± 0.5°C and 50%–70% relative humidity, with a 12:12‐hr light:dark photoperiod) following the protocol described by Ross, Axford, Richardson, Endersby‐Harshman, and Hoffmann (2017). This protocol is designed to reduce selection against individuals that are slow or quick to develop, mature, mate, blood feed, oviposit, or hatch, and to minimize mortality at each life stage. To maintain each population, all eggs from the previous generation were pooled and a random subset of larvae was provided with food (TetraMin^®^ tropical fish food tablets, Tetra, Melle, Germany) ad libitum and reared to adulthood. For the large populations, 400 adults were selected at random and added to 19.7‐L cages, while for the small populations and isofemale lines, 100 adults were added to 12‐L (30 × 20 × 20 cm) cages. For the inbred lines, a single male and female were added to a 1.5‐L (10 × 10 × 15 cm) cage. Except for the inbred lines, sex ratios were maintained naturally, and equal numbers of males and females were not counted. All cages were provided with a source of water and 10% sucrose. Approximately three days after the last adult had emerged, females were blood‐fed on a single human volunteer. Two days after blood feeding, cups containing larval rearing water and lined with sandpaper strips were introduced into the cages. Eggs laid on the sandpaper strips were collected over the span of 1 week, and all eggs were hatched three days after females had ceased oviposition. We followed this procedure until the Townsville populations were at F_13_, with each generation taking 28 days to complete. Blood feeding of mosquitoes on human subjects was approved by the University of Melbourne's Human Ethics Committee (approval #: 0723847). All volunteers provided informed written consent.

### 2.3 Fitness comparisons between Townsville F_13_ populations

We compared all Townsville populations at F_13_ for their development time and survival to adulthood under two nutrition conditions, and the fecundity and egg hatch rate of females reared under high nutrition conditions. Not all inbred lines were included in the experiments as the majority were lost by F_13_ (Supporting Information Table S2). Two of the four remaining inbred lines were only tested under high nutrition conditions due to low numbers, and these lines later became extinct (Supporting Information Table S2). Cairns (F_2_ and F_22_), Innisfail (F_4_ and F_10_), and Townsville (F_4/5_) populations were included in all experiments.

One hundred larvae from each population were reared in containers with 500 ml of water and provided with TetraMin^®^ ad libitum (high nutrition) or with 0.1 mg of TetraMin^®^ per larva every 2 days (low nutrition). Four replicate containers were reared for each population, except for two inbred lines where less than 400 larvae were obtained. A random subset of females from each population that emerged from the high nutrition treatment were blood‐fed and then isolated for oviposition. Eggs collected from each female were counted and hatched 3 days postcollection. Egg hatch rates were determined by calculating the proportion of eggs that had a detached cap.

Fitness data from the Townsville populations at F_13_ were used to estimate the performance of each population relative to the Townsville F_4/5_ ancestral population. We simplified an equation from Livdahl and Sugihara (1984) to calculate performance from fecundity, egg hatch, survival, and larval development time data. *F *is the mean fecundity of each population multiplied by egg hatch proportion, *S *is the mean proportion of larvae surviving to adulthood, and *D *is the mean larval development time in days. The performance index of each population at F_13_ was divided by the performance index of the ancestral population to determine their relative performance.

$$\text{Performance}\;\text{index}=\frac{\ln(F\times S)}{D}$$

### 2.4 Male mating competitiveness

We tested the male mating competitiveness of populations from Cairns that were at F_2_, F_7_, or F_27_ in the laboratory, and an inbred line from Townsville (Inbred A) at F_18_. Males from all populations competed against males infected with the *w*AlbB strain of *Wolbachia* for access to F_2_ females in a caged laboratory environment. Males infected with *w*AlbB induce complete sterility (eggs do not hatch) when crossed to uninfected females under standard laboratory conditions (Axford, Ross, Yeap, Callahan, & Hoffmann, 2016; Xi et al., 2005). Thus, the competitive ability of each population relative to *w*AlbB‐infected males can be estimated by scoring egg hatch rate from crosses between uninfected females and a mix of *Wolbachia*‐infected and uninfected males (Chambers et al., 2011; Segoli, Hoffmann, Lloyd, Omodei, & Ritchie, 2014). We established 12‐L cages containing 25 males from each population (F_2_, F_7_, F_27_, or inbred) and 25 males infected with *w*AlbB. Five replicate cages were established for each treatment. We then aspirated ten Cairns F_2_ females into each cage. This was repeated five times at 1‐hr intervals, for a total of 50 females per cage. Staggered releases were chosen to increase the level of male–male competition; adding all females to a cage at once would not provide many males with an opportunity to inseminate multiple females. All individuals were reared under the same conditions for this experiment (see *Colony maintenance*), and males were at least 24 hr old, and females at least 48 hr old before the sexes were combined. Females were blood‐fed 3 days after mating, and a single cup filled with larval rearing water and lined with a sandpaper strip was added to each cage. Sandpaper strips were collected daily and photographed, and the number of eggs on each strip was counted in ImageJ (Schneider, Rasband, & Eliceiri, 2012) using the Cell Counter plugin (https://imagej.nih.gov/ij/plugins/cell-counter.html). Eggs were hatched three days postcollection, and larvae were counted 3 days after hatching. Egg hatch rates were estimated by dividing the number of larvae counted by the number of eggs from each cage.

### 2.5 Pooled double‐digest RADseq library preparation

We used pooled double‐digest RADseq to determine the effective population size (*N*_e_) of the 17 replicate populations from Townsville at F_13_ relative to their ancestral population (F_1_). These included the five large populations, five small populations, five isofemale lines, and two inbred lines. We prepared a library following methods described by Rašić, Filipović, Weeks, and Hoffmann (2014) and Schmidt, Filipović, Hoffmann, and Rašić (2018) but modified the protocol for pooled mosquitoes. DNA was extracted from four pools of 20 adult mosquito heads from each population, with two pools for each sex, using a Roche DNA Isolation Kit for Cells and Tissues (Roche, Pleasanton, CA, USA). DNA from each pool was quantified using a Qubit dsDNA HS Assay Kit and a Qubit 2.0 Fluorometer (Thermo Fisher Scientific, Life Technologies Holdings Pte Ltd, Singapore), and the four pools for each population were combined after a normalization step.

750 ng of DNA from each population was digested in a 50 µl reaction with 20 units each of *Eco‐R1‐HF* and *SphI‐HF* restriction enzymes (New England Biolabs, Beverly, MA, USA), NEB CutSmart^®^ Buffer, and water for three hours at 37°C with no heat‐kill step. Restriction enzymes that cut less frequently were chosen to produce fewer SNPs while providing more coverage. The digestion products were cleaned with 75 µl of Ampure XP™ paramagnetic beads (Beckman Coulter, Brea, CA, USA), then ligated with modified Illumina P1 and P2 adapters overnight at 16°C with 1,000 units of T4 ligase and 1× T4 buffer (New England Biolabs) in a 45 µl reaction volume, and then heat deactivated for 10 min at 65°C. Ligations were cleaned using 75 µl of paramagnetic beads, and adapter‐ligated DNA fragments from all eighteen populations were pooled. We then used a Pippin Prep 2% gel cassette (Sage Sciences, Beverly, MA, USA) to select fragments with a size range of 350–450 bp. The final library was generated by pooling 38 10 µl PCRs and run for 12 cycles; each reaction contained 5 µl of Phusion High Fidelity 2× Master Mix (New England Biolabs), 2 µl each of 10 µM forward and reverse Illumina primers, and 2 µl of size‐selected DNA. The pooled PCRs were cleaned with 300 µl of paramagnetic beads, and a single library with DNA from 1,440 *Ae. aegypti* from the eighteen populations was sequenced in a single Illumina HiSeq 2000 lane to obtain 100 bp paired‐end reads.

### 2.6 Data processing and effective population size estimates

We checked the quality of the raw sequencing data with FastQC v0.11.5 (Andrews, 2010) and then used the process_radtags component of Stacks v1.46 (Catchen, Hohenlohe, Bassham, Amores, & Cresko, 2013) to demultiplex the populations, allowing for one mismatch. Reads were trimmed to 80 bp and then aligned to the *Aedes aegypti* reference genome AaegL4 (Dudchenko et al., 2017) using bowtie2 v2.3.0 (Langmead & Salzberg, 2012). Ambiguous alignments (minimum mapping quality below 20) were discarded, and alignments were converted to SAM format and sorted using SAMtools v1.4 (Li et al., 2009). Sorted files were then converted to mpileup format, with each file containing the ancestral population and one of the seventeen derived populations. These files were converted to sync format using the mpileup2sync.jar tool from Popoolation2 (Kofler, Pandey, & Schlotterer, 2011). We then estimated effective population size (*N*_e_) using the Nest R package v1.1.9 with three different methods (Jonas, Taus, Kosiol, Schlotterer, & Futschik, 2016).

### 2.7 Statistics on life history traits

All data were analyzed using SPSS Statistics version 24.0 for Windows (SPSS Inc, Chicago, IL, USA). Not all groups could be easily compared as variances across isofemale lines, and inbred lines were expected (and observed) to be much larger than the other populations. Data that were normally distributed were analyzed using general linear models (GLMs) and ANOVAs, and data that could not be normalized by transformations (log for development time and arcsine square root transformation for survival) were analyzed with Kruskal–Wallis and Mann–Whitney *U* tests. GLMs were used to investigate the effects of small and large populations for development time and survival, with sex included as a factor for development time and replicate cage nested within small and large populations. For development time, because of the very large impact of nutrition on this trait (approximately three times longer development on low nutrition), high and low nutrition conditions were considered separately, while for survival, a term for nutrition was included in the general linear model. Because multiple traits were compared for populations, we checked to see whether probabilities were still significant when adjusted for the number of traits by the Bonferroni procedure.

## 3 RESULTS

### 3.1 Preliminary fitness comparisons

When the Townsville population had reached F_2_, we compared life history traits to an established laboratory population from Cairns (F_11_); however, we observed no differences between populations for any trait (Supporting Information Appendix S1). At F_5_, we compared life history traits between replicated large populations and inbred lines. After two generations of brother–sister mating, the inbred lines had reduced fitness relative to the large populations, with substantial costs to larval survival and development time (Supporting Information Appendix S2). We also observed significant differences between replicate populations for some life history traits which likely arose due to founder effects or drift (Supporting Information Appendix S2). Fitness differences between populations can therefore arise after only a few generations.

### 3.2 Larval development time

**Figure 2:**
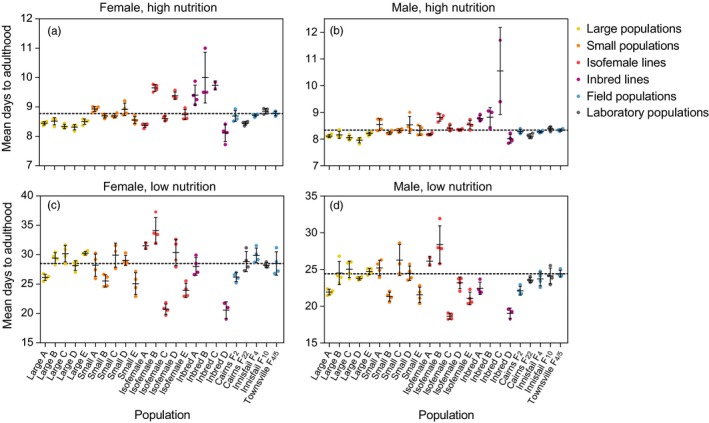
Development time of *Aedes aegypti* F_13_ laboratory populations maintained at different census sizes. Mean development time was measured for (a&c) female and (b&d) male larvae under (a&b) high nutrition (food provided ad libitum) and (c&d) low nutrition (0.1 mg of TetraMin^®^ per larva every 2 days) conditions. Each data point represents the mean development time of individuals from a single container of 100 larvae. Inbred lines B and C were not tested under low nutrition conditions. The dashed line represents the mean development time of the Townsville F_4/5 _ancestral population. Error bars are standard deviations

When the Townsville populations had reached F_13_, we performed fitness comparisons to test for inbreeding effects, laboratory adaptation, drift, and founder effects. We measured larval development time for all populations under high nutrition and low nutrition conditions (Figure 2). In the analysis of large and small populations under high nutrition conditions, there was a significant effect of sex, population, and replicate cage on (log) development time, but no interaction between sex and population or replicate cage (Table 1). Under low nutrition conditions, there was an effect of sex and replicate cage and an interaction between sex and population but no overall effect of population (Table 1). Small populations were developmentally delayed compared to large populations under high nutrition conditions but not low nutrition conditions (Figure 2). Under high nutrition conditions, significant differences in development time between replicate cages were also evident for isofemale lines (females: *F*_4,15_ = 81.888, *p* < 0.001; males: *F*_4,15_ = 18.956, *p* < 0.001) and inbred lines (females: *F*_3,9_ = 10.413, *p* = 0.003; males: *F*_3,9_ = 8.575, *p* = 0.005). The isofemale and inbred lines were particularly diverse; some lines performed as well as (or better than) the large populations, while others had greatly extended development times (Figure 2). Large populations (Townsville F_13_) were consistently faster to develop than the Townsville F_4/5_ ancestral population (females: *F*_1,22_ = 33.462, *p* < 0.001; males: *F*_1,22_ = 15.434, *p* = 0.001), suggesting a positive effect of laboratory maintenance on this trait. The Cairns F_22 _laboratory population was also faster to develop than the Cairns F_2_ field population (females: *F*_1,6_ = 6.407, *p* = 0.045; males: *F*_1,6_ = 9.147, *p* = 0.023), though the opposite was true for Innisfail, where the laboratory population was slower to develop (females: *F*_1,6_ = 6.653, *p* = 0.042; males: *F*_1,6_ = 5.938, *p* = 0.051); however, none of these *p* values were significant after Bonferroni adjustment. Under low nutrition conditions, development times were greatly extended relative to high nutrition conditions (Mann–Whitney *U*: *Z* = 16.250, *p* < 0.001, Figure 2). Under these conditions, the groups of populations maintained at different census sizes were less clearly distinguishable as there were significant differences between replicate populations at all census sizes (one‐way ANOVA: all *p* ≤ 0.002).

**Table 1:** General linear models on the large (census size 400) and small (census size 100) populations for (log) development time under high and low nutrition conditions, and on (arcsine square root) survival to adulthood

Trait	Source of variation	*df*	*F*	Significance
Development time (high nutrition)	Sex	1	318.492	<0.001
	Population	1	16.925	0.003
	Replicate cage (population)	8	15.070	<0.001
	Sex × population	1	0.676	0.435
	Sex × replicate (population)	8	0.453	0.884
Development time (low nutrition)	Sex	1	560.990	<0.001
	Population	1	0.430	0.530
	Replicate cage (population)	8	42.501	<0.001
	Sex × population	1	6.114	0.038
	Sex × replicate (population)	8	0.483	0.863
Survival to adulthood	Nutrition	1	19.370	0.002
	Population	1	22.547	0.001
	Replicate cage (population)	8	0.643	0.727
	Nutrition × population	1	1.547	0.249
	Nutrition × replicate (population)	8	1.549	0.160

### 3.3 Survival to adulthood and sex ratio

**Figure 3:**
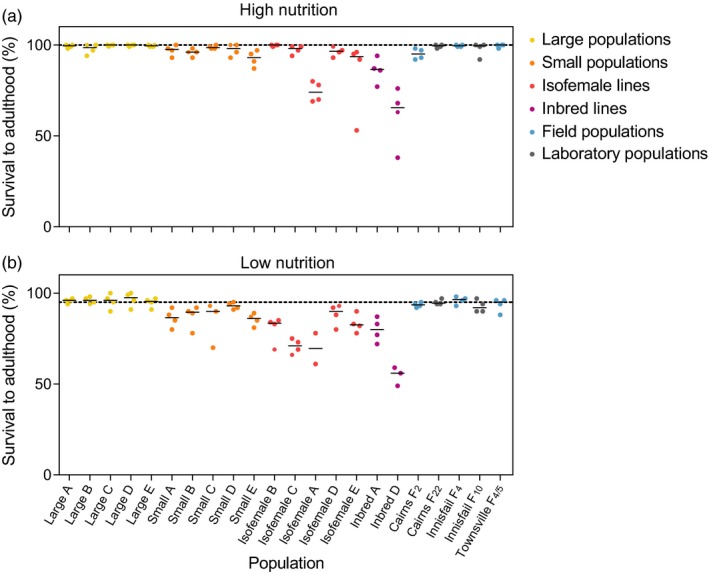
Survival to adulthood of *Aedes aegypti* F_13_ laboratory populations maintained at different census sizes. The percentage of larvae surviving to adulthood was tested under (a) high nutrition (food provided ad libitum) and (b) low nutrition (0.1 mg of TetraMin^®^ per larva every 2 days) conditions. Solid black lines indicate the median survival of each population. The dashed line represents the median survival of the Townsville F_4/5 _ancestral population

We compared the proportion of larvae that survived to adulthood between populations in the larval development experiment (Figure 3). Overall, there was an effect of nutrition and population on survival but no interaction effects when considering the large and small populations (Table 1). Under high nutrition conditions, survival rates approached 100% in most populations that were maintained at a census size of 400. Small populations had reduced survival rates compared to large populations (Table 1, Figure 3), as did the isofemale (Mann–Whitney *U*: *Z* = 3.489, *p* < 0.001) and inbred (*Z* = 4.018, *p* < 0.001) lines. We observed significant variation between isofemale lines (Kruskal–Wallis: χ^2^ = 14.872, *df* = 4, *p = *0.005), but not between large or small populations (Table 1, Figure 3). Survival to adulthood was poorer under low nutrition conditions (Mann–Whitney *U*: *Z* = 6.343, *p* < 0.001), but populations maintained at a census size of 400 still performed consistently better than populations maintained at lower census sizes (*Z* = 7.084, *p* < 0.001). No differences between laboratory and field populations from Townsville, Innisfail, or Cairns were evident (Mann–Whitney U: all *p* > 0.05), but the ability to detect any differences with this test was low due to low sample sizes for each population.

Sex ratios of adults emerging from the larval development experiment did not deviate significantly from 1:1 under high nutrition conditions for all populations (Chi‐square: *df* =3, all *p* > 0.05), except for the Cairns F_2_ population which was biased toward males (*df* =3, *p* = 0.013). Sex ratios were skewed toward males under low nutrition conditions (*df* =86, *p* < 0.001) which was likely the result of female larval mortality.

### 3.4 Fecundity and egg hatch rate

**Figure 4:**
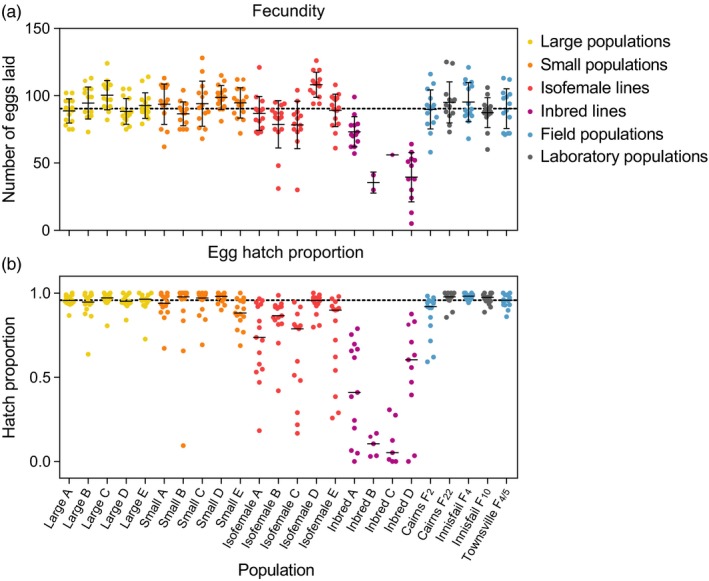
Fecundity (a) and egg hatch proportions (b) of *Aedes aegypti* F_13_ laboratory populations maintained at different census sizes. Fifteen females were tested per line, or as many as possible for inbred lines B and C. The dashed line represents the mean fecundity (a) or median egg hatch proportion (b) of the Townsville F_4/5 _ancestral population. Solid black lines indicate the mean fecundity (a) or median egg hatch proportion (b) of each population. Error bars are standard deviations

A random subset of females emerging from the larval development experiment was scored for their fecundity (Figure 4a) and egg hatch rate (Figure 4b). Inbred populations had greatly reduced fecundity compared to large populations (*F*_1,100_ = 130.395, *p* < 0.001). Replicate populations differed significantly from each other for large populations (*F*_4,69_ = 3.573, *p* = 0.010), isofemale lines (*F*_4,68_ = 10.300, *p* < 0.001), and inbred lines (*F*_3,24_ = 12.087, *p* < 0.001), but not small populations (*F*_4,66_ = 1.677, *p* = 0.166), potentially reflecting drift or founder effects. The fecundity of large populations (Townsville F_13_) did not differ from that of Townsville F_4/5_ (*F*_1,86_ = 0.549, *p* = 0.461), indicating that the effects of laboratory maintenance on this trait are minimal.

Egg hatch proportions were also substantially affected by inbreeding, with both isofemale lines (*Z* = 6.895, *p* < 0.001) and inbred lines (*Z* = 8.334, *p* < 0.001) exhibiting reduced hatch proportions relative to large populations (Figure 4b). There were differences between replicate populations for small populations (Kruskal–Wallis: χ^2^ = 10.405, *df* = 4, *p = *0.034), isofemale (χ^2^ = 19.639, *df *= 4, *p = *0.001), and inbred lines (χ^2^ = 11.222, *df* = 3, *p = *0.011), but not large populations (χ^2^ = 3.141, *df* = 4, *p = *0.535). Hatch proportions did not differ between the Townsville F_4/5_ population and the large populations at F_13 _(Mann–Whitney *U*: Z = 0.2137, *p* = 0.834), but were improved in the Cairns F_22_ population relative to Cairns F_2_ (*Z* = 3.202, *p* = 0.001), suggesting a positive effect of laboratory maintenance.

### 3.5 Overall performance

**Figure 5:**
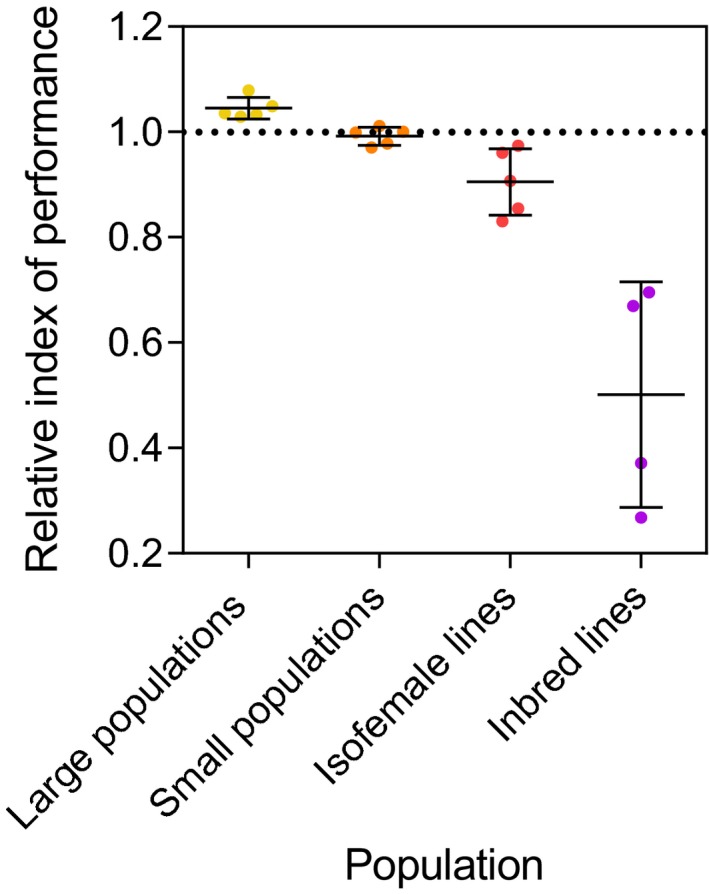
Relative performance of *Aedes aegypti* F_13_ laboratory populations maintained at different census sizes. Each data point represents the performance index of a single replicate population relative to the ancestral population (Townsville F_4/5_) which is represented by the black dotted line. Black bars indicate means and standard deviations

We calculated an index of performance for each Townsville population at F_13_ relative to the ancestral population (Townsville F_4/5_) using the data for fecundity, egg hatch proportion, larval development time, and survival to adulthood (under high nutrition conditions) available for each population (Figure 5). The large populations (census size 400) consistently performed better than the ancestral population (one‐sample *t* test, *p* = 0.008) which indicates a positive effect of artificial rearing conditions on performance in the laboratory. The increased performance of laboratory populations was largely due to shorter larval development time (Supporting Information Appendix S3). The relative performance of populations declined substantially with increasing levels of inbreeding; isofemale lines and inbred lines had much poorer performance than the ancestral population. This fitness deficit could largely be restored by crossing inbred mosquitoes to an outbred population (Supporting Information Appendix S4). The Cairns laboratory population (F_22_) had increased performance over the field (F_2_) population (relative performance index: 1.080), but the Innisfail laboratory population (F_10_) had decreased performance over the field population (F_4_) (relative performance index: 0.960). Laboratory populations therefore did not always exhibit increased performance over the populations that were colonized more recently.

### 3.6 Effective population size

We estimated the effective population size (*N*_e_) of the replicate Townsville populations at F_13_ relative to the ancestral population (F_1_) using pooled RADseq and the Nest R package v1.1.9 (Jonas et al., 2016). The *N_e_(JR)* and *N_e_(P)* methods provided similar estimates of *N*_e_, but *N_e_(W)* provided estimates that were in many cases much larger than the census sizes. For estimates calculated using the *N_e_(JR)* and *N_e_(P)* methods, *N*_e_ declined substantially with decreasing census size (Table 2). Ratios of *N*_e_ to census size calculated using the *N_e_(P)* method were low, though the small populations (census size 100, mean *N*_e_/*N* = 0.250) had higher ratios than large populations (census size 400, mean *N*_e_/*N* = 0.143). The index of performance for each population increased dramatically with increasing *N*_e_ but levelled off at higher *N*_e_ (Supporting Information Figure S1). These findings demonstrate a clear association between *N*_e_ and fitness (Spearman's rank‐order correlation: ρ = 0.973, *p* < 0.001, *n* = 17) but suggests that an *N*_e_ greater than used in the large populations will lead to only small fitness improvements.

**Table 2:** Effective population sizes (*N*_e_) of *Aedes aegypti* F_13_ laboratory populations maintained at different census sizes, calculated using three temporal methods

Population	Replicate	*N*_e_ estimate		
		*N_e_*(*W*) (Waples, 1989)	*N_e_*(*JR*) (Jorde & Ryman, 2007)	*N_e_*(*P*) (Jonas et al., 2016)
Large populations (*N* = 400)	A	661.073	71.566	55.827
	B	903.292	55.903	53.776
	C	568.557	74.208	60.054
	D	846.441	73.280	60.850
	E	1,048.192	65.394	55.632
Small populations (*N* = 100)	A	526.797	24.428	24.435
	B	190.602	20.666	17.814
	C	761.790	28.871	34.351
	D	183.127	22.279	20.863
	E	162.276	24.785	22.982
Isofemale lines	A	191.258	10.015	11.437
	B	182.663	10.366	9.782
	C	154.080	9.541	9.038
	D	188.197	11.173	11.507
	E	148.858	10.139	9.694
Inbred lines	A	271.954	4.213	5.184
	E	323.245	4.610	5.408

### 3.7 Mating competitiveness

**Figure 6:**
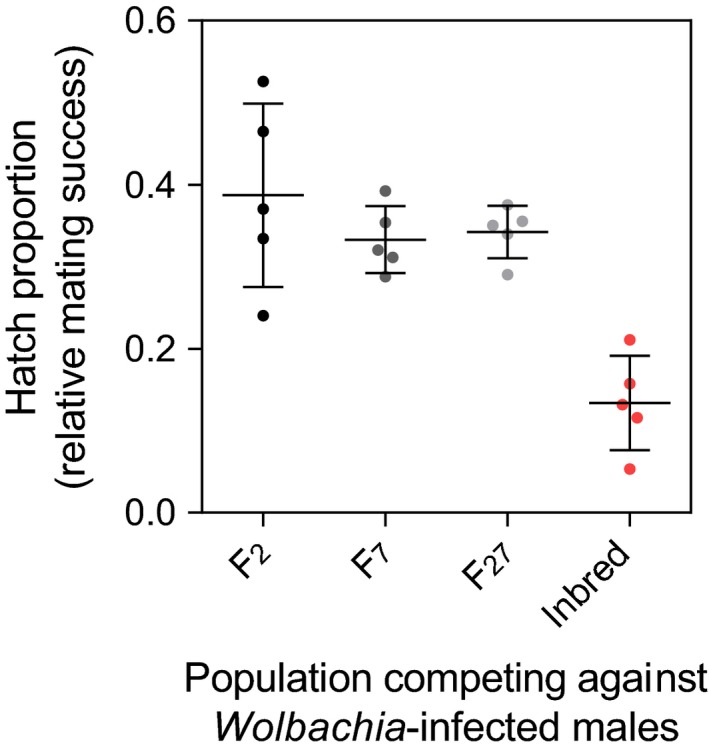
Relative mating success of *Aedes aegypti* males maintained in the laboratory for different numbers of generations. We tested the relative mating success of males from Cairns F_2_, F_7_, and F_27_ populations when competing against *Wolbachia*‐infected males for access to Cairns F_2_ females. An inbred colony (Inbred A F_18_) was included for comparison. Higher hatch proportions indicate increased mating success of the experimental males relative to *Wolbachia*‐infected males. Each data point represents the mean egg hatch proportion from a cage of 50 females. Black bars indicate means and standard deviations

Males from the Cairns F_2_, F_7_, or F_27_ and inbred (Inbred A F_18_) populations competed for access to F_2_ females against a standard competitor infected with *Wolbachia *(*w*AlbB strain) (Figure 6). Hatch proportions did not differ significantly between the F_2_, F_7_, and F_27 _populations (one‐way ANOVA: *F*_2,12_ = 0.829, *p* = 0.460), but were markedly reduced for inbred males relative to the other populations (one‐way ANOVA: *F*_1,18_ = 39.784, *p* < 0.001). These results indicate that male mating success in laboratory cages is not affected by long‐term laboratory maintenance, but can be decreased by inbreeding. The poor performance of inbred males was likely due to reduced mating success and not a paternal effect on female fertility, as crosses between inbred males and Cairns F_2_ females produced eggs with high hatch proportions (Supporting Information Appendix S4).

## 4 DISCUSSION

We performed a comprehensive assessment of inbreeding and laboratory adaptation in *Ae. aegypti* mosquitoes to inform rear‐and‐release programs for arbovirus control. Our study is the first to investigate the effects of inbreeding on *Ae. aegypti* fitness directly by comparing outbred and inbred lines derived from the same population, and the first that links fitness costs to reductions in effective population size as assessed through genomic markers. We look for evidence of adaptation by comparing laboratory populations to their direct ancestor concurrently and use replicate populations to separate fitness changes due to adaptation from drift and founder effects, two approaches which have not been previously applied in mosquitoes.

We find evidence of laboratory adaptation in colonized *Ae. aegypti* populations, but changes in trait means were small in magnitude and directions were often inconsistent between populations. All replicate laboratory populations from Townsville developed faster and were smaller than mosquitoes from the ancestral population. These changes could be a response to selection for abbreviated development in the laboratory, despite efforts to avoid selection in our laboratory rearing protocol (Ross et al., 2017). Shorter developmental periods are often observed in laboratory‐adapted insects (Allgood & Yee, 2014), particularly under mass‐rearing conditions that favor the rapid production of insects (Economopoulos, 1992; Miyatake, 1993). In contrast, development times can increase in colonized *Drosophila* maintained with overlapping generations, where there is less selection against slow developing individuals (Sgro & Partridge, 2000). Apart from size and development time, laboratory populations of *Ae. aegypti *were generally representative of field populations for most traits after one year in the laboratory. However, rearing mosquitoes on a larger scale may introduce additional selective pressures that affect field performance resulting from crowded rearing conditions (Zhang et al., 2018).

When fitness traits were combined into an overall index of performance, we found that laboratory populations maintained at a large census size usually had greater performance than field populations. This finding is consistent with other insects, where fitness under laboratory conditions tends to improve with laboratory maintenance (Hoffmann & Ross, 2018), though a recent review and set of experiments in *Drosophila* found a lack of clear directional trends across multiple species (Maclean, Kristensen, Sorensen, & Overgaard, 2018). The rate of adaptation in our laboratory colonies of *Ae. aegypti* was slower than other mosquito species and insects in general (Hoffmann & Ross, 2018). *Aedes aegypti* collected from the field performed well from the first generation in the laboratory, potentially because this species is already somewhat adapted to living in artificial environments (Cheong, 1967). Rates of adaptation are likely to be higher for species such as *Aedes notoscriptus* where the laboratory environment is suboptimal and only a small proportion of individuals can reproduce in the initial generations (Watson et al., 2000). Populations tested at F_2_ did not tend to differ from laboratory populations in terms of trait means, but some traits exhibited greater variation at F_2_. This suggests that *Ae. aegypti* could lose variation with laboratory maintenance, though other traits for other populations at F_2_ had similar variances to laboratory populations.

A limitation of our experiments is that we assessed the effects of inbreeding and laboratory adaptation under laboratory conditions. High fitness under these conditions does not necessarily indicate high fitness in the field (Kristensen, Loeschcke, & Hoffmann, 2007; Thomson & Hoffmann, 2002); therefore, the apparent lack of laboratory adaptation observed here might not translate to the field where conditions are more complex. Several factors may also confound the results of our experiments. Our main comparisons were between populations at F_4/5_ and F_13_; if substantial laboratory adaptation occurs then we would be unable to detect it with these comparisons. Our population comparisons could also be confounded by selection on the ancestral population due to eggs experiencing quiescence (Townsville and Innisfail populations) or differences present in populations collected from the same location but at different times (Cairns populations). Other factors such as gut microbiota could also confound our comparisons between laboratory and field populations because the microbiome can greatly influence mosquito life history traits (Coon, Brown, & Strand, 2016; Coon, Vogel, Brown, & Strand, 2014). Gut microbiota are much less diverse in colonized mosquitoes (Mwadondo, Ghilamicael, Alakonya, & Kasili, 2017) and tend to be similar in laboratory populations regardless of geographic origin (Dickson et al., 2017). This could be an issue when comparing field and laboratory populations.

Few studies on laboratory adaptation in insects attempt to separate the effects of laboratory adaptation from drift or founder effects (Hoffmann et al., 2001; is one exception) which are likely to be substantial when establishing small laboratory colonies. We used replicate populations to avoid this issue; consistent divergence in colonized populations from the ancestral population indicates adaptation, variation between replicate populations immediately after establishment indicates founder effects, and divergence between replicate populations at the time indicates drift. We found that replicate populations at the same census size differ significantly from each other for several fitness traits, both at F_5_ and at F_13_, particularly for populations maintained at low census sizes. Fitness differences between replicate populations were not always consistent between F_5_ and F_13_, suggesting that both founder effects and drift occur. These findings are of concern for laboratory studies that compare traits between populations maintained separately. Researchers should consider using replicate populations when conducting experiments or outcross populations frequently to maintain similar genetic backgrounds (Yeap et al., 2011).

We demonstrate that inbreeding is extremely costly to *Ae. aegypti *fitness. Most inbred lines were lost across the experiment, and the remaining lines performed substantially worse than outbred populations. Relatively few studies have specifically addressed the effects of inbreeding on mosquito fitness. Powell and Evans (2016) observed that inbreeding *Ae. aegypti* through full‐sib mating reduces heterozygosity by much less than expected based on theory, and deleterious recessive alleles must therefore be common. Koenraadt, Kormaksson, and Harrington (2010) reported fitness costs of inbred *Ae. aegypti* larvae relative to a wild population, and inbreeding through full‐sib mating reduces fitness in other *Aedes* species (Armbruster, Hutchinson, & Linvell, 2000; O'Donnell & Armbruster, 2010). We demonstrate that mosquito populations inbred intentionally, for instance, to generate homozygous transgenic strains (Catteruccia, Godfray, & Crisanti, 2003; Phuc et al., 2007), will likely suffer from severe inbreeding depression. However, it may be possible to retain partial fitness if there is also selection for certain life history traits during inbreeding (Shetty et al., 2016). We show that laboratory populations maintained at low census sizes (*N* = 100) also experience inbreeding depression, and the loss of fitness correlates strongly with decreased effective population size. Thus, laboratories should ensure that population sizes in colonized mosquitoes are sufficiently high to maintain their fitness. Our laboratory populations for these experiments were each established from only a few hundred individuals, and we would recommend that larger numbers be used to avoid bottlenecks.

Our laboratory populations at F_13_ had a substantially lower *N*_e_ than field populations from Townsville (Endersby et al., 2011) and other locations around the world (Saarman et al., 2017). However, ratios of *N*_e_ to census size (*N*_e_/*N*) were similar to ratios reported in nature (Saarman et al., 2017). *N*_e_/*N* ratios were larger in the small laboratory populations (*N* = 100) than in the large ones (*N* = 400), consistent with a study of *Drosophila* populations maintained at different census sizes (Schou, Loeschcke, Bechsgaard, Schlotterer, & Kristensen, 2017). Low *N*_e_/*N* ratios indicate that reproductive success varies greatly between individuals (Hedrick, 2005; Nunney, 1995), and this appears to be the case for large colonized populations of *Ae. aegypti*. Unequal contributions to the next generation occur because we sample only a few hundred individuals randomly from a pool of thousands of larvae, and we do not equalize offspring from each female to establish the next generation (Ross et al., 2017).

We demonstrate that the consequences of laboratory maintenance in *Ae. aegypti* can be minimized by maintaining large population sizes, but there are several other ways to maintain the fitness of colonized mosquito populations. The simplest approach is to cross laboratory colonies to an outbred population (Yeap et al., 2011). Gene flow into inbred populations commonly leads to a fitness improvement (Frankham, 2015), and we also show that the fitness of inbred *Ae. aegypti* can be improved through a single generation of outcrossing. Increased performance of hybrids has been demonstrated in *Anopheles* mosquitoes (Baeshen et al., 2014; Ekechukwu et al., 2015; Menge et al., 2005) and the Queensland fruit fly (Gilchrist & Meats, 2014), with fitness improvements in the F_1_. Crosses between different laboratory lines can also be used to determine whether changes in fitness in laboratory populations are due to inbreeding or adaptation (Baeshen et al., 2014). Rates of laboratory adaptation can be slowed by using more natural rearing environments. Knop et al. (1987) compared two methods of rearing *Culex tarsalis* and found that colonies maintained in larger cages at a variable temperature and more complex environmental conditions had a slower rate of laboratory adaptation. Ng'habi et al. (2015) found that rearing *Anopheles arabiensis* under semi‐field conditions preserved their similarity to the wild population and reduced the extent of inbreeding. Quality control methods such as screening mosquitoes for their flight capacity can also be used to increase fitness before their deployment for disease control programs (Balestrino, Puggioli, Carrieri, Bouyer, & Bellini, 2017).

In summary, we provide evidence for inbreeding depression effects and a small effective population size relative to census size in laboratory mosquito populations, along with some limited laboratory adaptation particularly in large populations. Our results have implications for the maintenance of insects in the laboratory, particularly for those destined for open field releases. While we find that life history traits of *Ae. aegypti* do not change consistently with laboratory maintenance, traits where selective pressures are absent in the laboratory, such as flight ability, feeding behavior, and thermal tolerance, might still be compromised.

## CONFLICT OF INTEREST

None declared.

## Data Availability

Data for this study are available at the Dryad Digital Repository: https://doi.org/10.5061/dryad.84q8c68.
