# Supplementary material for: A comprehensive assessment of inbreeding and laboratory adaptation in Aedes aegypti mosquitoes
Source: Evol Appl. 2018 Dec 17;12(3):572–86. doi: 10.1111/eva.12740 (PMC6383739; doi:10.1111/eva.12740)
Supplement: Supplementary file 2 [file EVA-12-572-s002.docx]

**S1 Table.** Studies of laboratory adaptation in mosquitoes. Studies compared mosquito populations that were maintained in the laboratory for different numbers of generations. Focal traits were either tested over successive generations in the laboratory, or field and laboratory populations were compared simultaneously.

| **Species** | **Focal trait** | **Comparison** | **Effect of laboratory maintenance** | **Reference** |
| --- | --- | --- | --- | --- |
| *Aedes aegypti* | Blood feeding duration | Tracked changes over 4 generations | Reduced blood feeding duration | (Chadee and Beier 1997; Chadee, Beier, and Mohammed 2002) |
| *Aedes aegypti* | Susceptibility to yellow fever | Tracked changes over 11 generations | Significant differences between generations | (Lorenz et al. 1984) |
| *Aedes aegypti* | Susceptibility to dengue | Field population *vs* laboratory population | Reduced susceptibility to dengue | (Salazar et al. 2007) |
| *Aedes aegypti* | Quiescent egg viability | Field population (G1) *vs* laboratory population (G60) | No effect on egg viability under almost all conditions | (Faull and Williams 2015) |
| *Aedes aegypti* | Sperm quantity | Field population (G0) *vs* two laboratory populations | Reduced quantity of sperm in both laboratory populations | (Ponlawat and Harrington 2007) |
| *Aedes aegypti* | Wing shape | Field population (G1) *vs* laboratory population (G8) | Altered wing shape | (Yeap et al. 2013) |
| *Aedes aegypti* | Copulation and insemination rate, male body size | Field population (G1) *vs* laboratory population (~G40) | Decreased copulation and insemination rate, decreased body size | (Richardson and Williams 2013) |
| *Aedes aegypti* | Susceptibility to permethrin | Field population (G0) *vs* laboratory population (G10) | Increased susceptibility to permethrin | (Grossmann et al. 2018) |
| *Aedes albopictus* | Development time and survival to adulthood, under different levels of nutrition | Field population (G2) *vs* laboratory population (G>300) | No effect, except under low nutrition conditions where the field population has higher survival to adulthood | (Jong et al. 2017) |
| *Aedes albopictus* | Susceptibility to dengue virus | Field populations (G<5) *vs* laboratory populations (G>40) | Increased susceptibility | (Vazeille et al. 2003) |
| *Aedes notoscriptus* | Egg hatch rate | Tracked changes over 13 generations | Increased egg hatch rate (likely due to increased mating success) | (Watson, Marshall, and Kay 2000) |
| *Aedes triseriatus* | Susceptibility to La Crosse virus | Various field and laboratory populations | Varied effect on susceptibility | (Grimstad et al. 1977) |
| *Culex tarsalis* | Swarming behavior and insemination rate (females) | Field populations (G0) *vs* laboratory population | Altered swarming behaviour and increased female insemination rate | (Reisen, Knop, and Peloquin 1985) |
| *Culex tarsalis* | Levels of essential fatty acids | Various field and laboratory populations | Reduced levels of triacylglycerol eicosapentaenoic acid and triacylglycerol arachidonic acid | (Dadd, Kleinjan, and Asman 1988) |
| *Culex tarsalis* | Insemination rate (females), mating competitiveness, blood feeding success, oviposition success | Tracked changes over 12 generations, compared various laboratory populations maintained with different rearing conditions | Increased insemination success, mating competitiveness, blood feeding success and oviposition success | (Knop et al. 1987) |
| *Culex quinquefasciatus* | Development time, survival to adulthood and body size | Field population (G2) *vs* laboratory population | Faster development time, increased survival to adulthood, larger size | (Allgood and Yee 2014) |
| *Culex quinquefasciatus* | Oviposition behaviour | Field population (G3) *vs* laboratory population | Altered oviposition behaviour | (Allgood and Yee 2017) |
| *Culex nigripalpus* | Insemination rate (females) | Field population (G1) *vs* laboratory population (G54) | Increased insemination success (independent of male strain) | (Haeger and O'Meara 1970) |
| *Anopheles albitarsis* | Insemination rate (males) | Field population (G1) *vs* laboratory population (G150) | Increased insemination success (independent of female strain) | (Lima, Valle, and Peixoto 2004) |
| *Anopheles arabiensis* | Insemination rate and recapture rate (males) | Field population (G0) *vs* laboratory population (G68) | No change in insemination rate or recapture rate | (Hassan et al. 2010) |
| *Anopheles arabiensis* | Rate of male sexual maturation | Field population (G0) *vs* laboratory population (G125) | Increased rate of sexual maturation | (Oliva et al. 2011) |
| *Anopheles arabiensis* | Abundance of energetic reserves (males), wing size (males) | Field population (G0) *vs* laboratory population (G10) | Increased glycogen and glucose abundance, reduced lipid abundance, reduced wing size | (Ng'habi et al. 2015) |
| *Anopheles coluzzii* | Quantity of sperm and mating plug proteins, insemination rate, reproductive success, longevity | Field population (G1) *vs* two laboratory populations (8 y and >35 y) | Substantial changes in several mating traits | (Ekechukwu et al. 2015) |
| *Anopheles darlingi* | Fecundity, egg hatch rate, adult emergence and susceptibility to *Plasmodium vivax* | Tracked changes over 5 generations | No change in life history traits, no change in susceptibility to *Plasmodium vivax* | (Moreno et al. 2014) |
| *Anopheles gambiae* | Energetic reserves and body size of males | Field population (G0) *vs* laboratory population | Reduced body size and lipid reserves | (Huho et al. 2007) |
| *Anopheles gambiae* | Testes size, accessory gland size | Field population (G1) *vs* laboratory populations | Increased testes size, decreased accessory gland size | (Baeshen et al. 2014) |

*Literature cited*

Allgood, D. W. and D. A. Yee. 2014. Influence of resource levels, organic compounds and laboratory colonization on interspecific competition between the Asian tiger mosquito *Aedes albopictus* (*Stegomyia albopicta*) and the southern house mosquito *Culex quinquefasciatus*. *Medical and Veterinary Entomology* 28 (3):273-86.

Allgood, David W. and Donald A. Yee. 2017. Oviposition preference and offspring performance in container breeding mosquitoes: evaluating the effects of organic compounds and laboratory colonisation. *Ecological Entomology*.

Baeshen, R., N. E. Ekechukwu, M. Toure, D. Paton, M. Coulibaly, S. F. Traore, and F. Tripet. 2014. Differential effects of inbreeding and selection on male reproductive phenotype associated with the colonization and laboratory maintenance of *Anopheles gambiae*. *Malar. J.* 13 (1):19.

Chadee, D. D. and J. C. Beier. 1997. Factors influencing the duration of blood-feeding by laboratory-reared and wild *Aedes aegypti* (Diptera: Culicidae) from Trinidad, West Indies. *Annals of Tropical Medicine and Parasitology* 91 (2):199-207.

Chadee, D. D., J. C. Beier, and R. T. Mohammed. 2002. Fast and slow blood-feeding durations of *Aedes aegypti* mosquitoes in Trinidad. *Journal of Vector Ecology* 27 (2):172-7.

Dadd, R. H., J. E. Kleinjan, and S. M. Asman. 1988. Eicosapentaenoic acid in mosquito tissues: differences between wild and laboratory-reared adults. *Environmental Entomology* 17 (2):172-180.

Ekechukwu, N. E., R. Baeshen, S. F. Traore, M. Coulibaly, A. Diabate, F. Catteruccia, and F. Tripet. 2015. Heterosis increases fertility, fecundity, and survival of laboratory-produced F1 hybrid males of the malaria mosquito *Anopheles coluzzii*. *G3 (Bethesda)* 5 (12):2693-709.

Faull, K. J. and C. R. Williams. 2015. Intraspecific variation in desiccation survival time of *Aedes aegypti* (L.) mosquito eggs of Australian origin. *Journal of Vector Ecology* 40 (2):292-300.

Grimstad, Paul R, George B Craig Jr, Quentin E Ross, and Thomas M Yuill. 1977. *Aedes triseriatus* and La Crosse virus: geographic variation in vector susceptibility and ability to transmit. *Am J Trop Med Hyg* 26 (5):990-996.

Grossmann, M. K., V. Uc-Puc, J. Rodriguez, D. J. Cutler, L. T. Morran, P. Manrique-Saide, and G. M. Vazquez-Prokopec. 2018. Restoration of pyrethroid susceptibility in a highly resistant *Aedes aegypti* population. *Biol Lett* 14: 20180022.

Haeger, James S and George F O'Meara. 1970. Rapid incorporation of wild genotypes of *Culex nigripalpus* (Diptera: Culicidae) into laboratory-adapted strains. *Annals of the Entomological Society of America* 63 (5):1390-1391.

Hassan, Mo’awia M, Waleed M El-Motasim, Rania T Ahmed, and Badria B El-Sayed. 2010. Prolonged colonisation, irradiation, and transportation do not impede mating vigour and competitiveness of male *Anopheles arabiensis* mosquitoes under semi-field conditions in Northern Sudan.

Huho, B. J., K. R. Ng'habi, G. F. Killeen, G. Nkwengulila, B. G. Knols, and H. M. Ferguson. 2007. Nature beats nurture: a case study of the physiological fitness of free-living and laboratory-reared male *Anopheles gambiae* s.l. *J Exp Biol* 210 (Pt 16):2939-47.

Jong, Z. W., N. F. A. Kassim, M. A. Naziri, and C. E. Webb. 2017. The effect of inbreeding and larval feeding regime on immature development of *Aedes albopictus*. *J. Vector Ecol.* 42 (1):105-112.

Knop, Nancy Fike, S Monica Asman, William K Reisen, and Marilyn M Milby. 1987. Changes in the biology of *Culex tarsalis* (Diptera: Culicidae) associated with colonization under contrasting regimes. *Environmental Entomology* 16 (2):405-414.

Lima, J. B., D. Valle, and A. A. Peixoto. 2004. Adaptation of a South American malaria vector to laboratory colonization suggests faster-male evolution for mating ability. *BMC evolutionary biology* 4 (1):12.

Lorenz, L., B. J. Beaty, T. H. G. Aitken, G. P. Wallis, and W. J. Tabachnick. 1984. The effect of colonization upon *Aedes aegypti* - susceptibility to oral infection with yellow fever virus. *American Journal of Tropical Medicine and Hygiene* 33 (4):690-694.

Moreno, M., C. Tong, M. Guzman, R. Chuquiyauri, A. Llanos-Cuentas, H. Rodriguez, D. Gamboa, S. Meister, E. A. Winzeler, P. Maguina, J. E. Conn, and J. M. Vinetz. 2014. Infection of laboratory-colonized *Anopheles darlingi* mosquitoes by *Plasmodium vivax*. *The American Journal of Tropical Medicine and Hygiene* 90 (4):612-6.

Ng'habi, K. R., Y. Lee, B. G. Knols, D. Mwasheshi, G. C. Lanzaro, and H. M. Ferguson. 2015. Colonization of malaria vectors under semi-field conditions as a strategy for maintaining genetic and phenotypic similarity with wild populations. *Malar J* 14:10.

Oliva, Clelia F, Mark Q Benedict, Guy Lempérière, and Jérémie Gilles. 2011. Laboratory selection for an accelerated mosquito sexual development rate. *Malar J* 10 (1):135.

Ponlawat, Alongkot and Laura C Harrington. 2007. Age and body size influence male sperm capacity of the dengue vector *Aedes aegypti* (Diptera: Culicidae). *J Med Ent* 44 (3):422-426.

Reisen, William K, Nancy F Knop, and John J Peloquin. 1985. Swarming and mating behavior of laboratory and field strains of *Culex tarsalis* (Diptera: Culicidae). *Annals of the Entomological Society of America* 78 (5):667-673.

Richardson, A. J. and C. R. Williams. 2013. Inter-population mating success in Australian dengue vector mosquitoes: effects of laboratory colonization and implications for the spread of transgenics. *J Vector Ecol* 38 (1):111-119.

Salazar, M. I., J. H. Richardson, I. Sanchez-Vargas, K. E. Olson, and B. J. Beaty. 2007. Dengue virus type 2: replication and tropisms in orally infected *Aedes aegypti* mosquitoes. *BMC Microbiol* 7:9.

Vazeille, Marie, Leon Rosen, Laurence Mousson, and Anna-Bella Failloux. 2003. Low oral receptivity for dengue type 2 viruses of *Aedes albopictus* from Southeast Asia compared with that of *Aedes aegypti*. *Am J Trop Med Hyg* 68 (2):203-208.

Watson, T. M., K. L. Marshall, and B. H. Kay. 2000. Colonization and laboratory biology of *Aedes notoscriptus* from Brisbane, Australia. *Journal of the American Mosquito Control Association* 16 (2):138-42.

Yeap, H. L., N. M. Endersby, P. H. Johnson, S. A. Ritchie, and A. A. Hoffmann. 2013. Body size and wing shape measurements as quality indicators of *Aedes aegypti* mosquitoes destined for field release. *Am J Trop Med Hyg* 89 (1):78-92.
