# Supplementary material for: A comprehensive assessment of inbreeding and laboratory adaptation in Aedes aegypti mosquitoes
Source: Evol Appl. 2018 Dec 17;12(3):572–86. doi: 10.1111/eva.12740 (PMC6383739; doi:10.1111/eva.12740)
Supplement: Supplementary file 3 [file EVA-12-572-s003.docx]

**S2 Table.** Maintenance and loss of inbred *Aedes aegypti* populations during successive generations of full-sib mating.

| **Generation in the laboratory** | **Generations of full-sib mating** | **Inbred lines remaining** | **Replicate pairs maintained for each line*** |
| --- | --- | --- | --- |
| F_3_ | 0 | 10 | 2 |
| F_4_ | 1 | 10 | 2 |
| F_5_ | 2 | 10 | 2 |
| F_6_ | 3 | 9 | 2 |
| F_7_ | 4 | 7 | 4 |
| F_8_ | 5 | 4 | 6 |
| F_9_ | 6 | 4 | 6 |
| F_10_ | 7 | 4 | 6 |
| F_11_ | 8 | 4 | 10 |
| F_12_ | 9 | 4 | 10 |
| F_13_ | 9 | 4 | All offspring interbred for each line |
| F_14_ | 9 | 2 | All offspring interbred for each line |

* The number of replicate male and female pairs for each line increased as inbred lines were lost.
