# Supplementary material for: A comprehensive assessment of inbreeding and laboratory adaptation in Aedes aegypti mosquitoes
Source: Evol Appl. 2018 Dec 17;12(3):572–86. doi: 10.1111/eva.12740 (PMC6383739; doi:10.1111/eva.12740)
Supplement: Supplementary file 4 [file EVA-12-572-s004.docx]

**S1 Appendix.** Pilot experiments comparing fitness differences between Townsville F_2_ and Cairns F_11_ *Aedes aegypti* populations.

*Materials and Methods*

We conducted a series of experiments on the Townsville population at F_2_ by comparing a range of fitness traits to an established laboratory population from Cairns (F_11_). Adult longevity was compared using methods described by Axford et al. (2016). In short, eight replicate cages containing 25 females and 25 males for each population were checked three times per week for mortality until all adults had died. Adults were provided with a sucrose solution and a source of water, and females were blood-fed weekly. Larval development time was also assessed under two levels of nutrition. Eight replicate containers with 50 larvae each in 200 mL of water were provided with TetraMin *ad libitum* (high nutrition) or with 0.1 mg of TetraMin per larva every 2 days (low nutrition). Adult emergence was scored twice daily (high nutrition) or daily (low nutrition) until all individuals had completed development or died. The survival of larvae under starvation conditions was compared using methods described by Ross et al. (2016). Larvae were provided with food *ad libitum* for 72 hr and then transferred to reverse osmosis (RO) water with no food provided. Larvae were either held in containers of 200 mL of water in groups of 50 (starvation survival in groups) or individual larvae were held in wells of 12-well cell culture plates containing 4 mL of water (starvation survival in isolation). The number of larvae alive was then scored every two days until all larvae had died. For starvation survival in groups, two independent experiments were conducted with eight replicates each. For the individual experiment, 144 isolated larvae were tested per population. Fecundity was also scored following the methods of Ross and others (2016) over three gonotrophic cycles. Females were blood fed *en masse* and 30 females per population were isolated in 70 mL cups containing a moist sandpaper oviposition substrate. Sandpaper strips were collected five days after blood feeding and the number of eggs was counted using a clicker counter. Females were blood fed one and two weeks after the initial blood feeding to initiate further gonotrophic cycles. Survival data for adult longevity and larval starvation experiments were investigated using Kaplan-Meier analysis, with log-rank tests comparing rates of mortality between populations.

*Results*

We compared the Townsville F_2_ population to the Cairns F_11_ population. We observed no clear differences between the two populations for all traits tested (Figure 1). Development time under two nutrition conditions (one-way ANOVA: F_1,50_ = 0.150, P = 0.700), fecundity across three gonotrophic cycles (F_1,118_ = 2.568, P = 0.112) and the longevity of females (Kaplan-Meier: χ^2^ = 1.592, df = 1, P = 0.207) and males (χ^2^ = 3.725, df = 1, P = 0.054) were not significantly different between the two populations. The two populations also did not differ in their ability to survive under starvation conditions, both when larvae were held in groups (χ^2^ = 0.670, df = 1, P = 0.413) and in isolation (χ^2^ = 3.093, df = 1, P = 0.079). While no clear differences were observed, the two populations were derived from locations 350 km apart, and differences between the original field populations could affect the results of experiments.


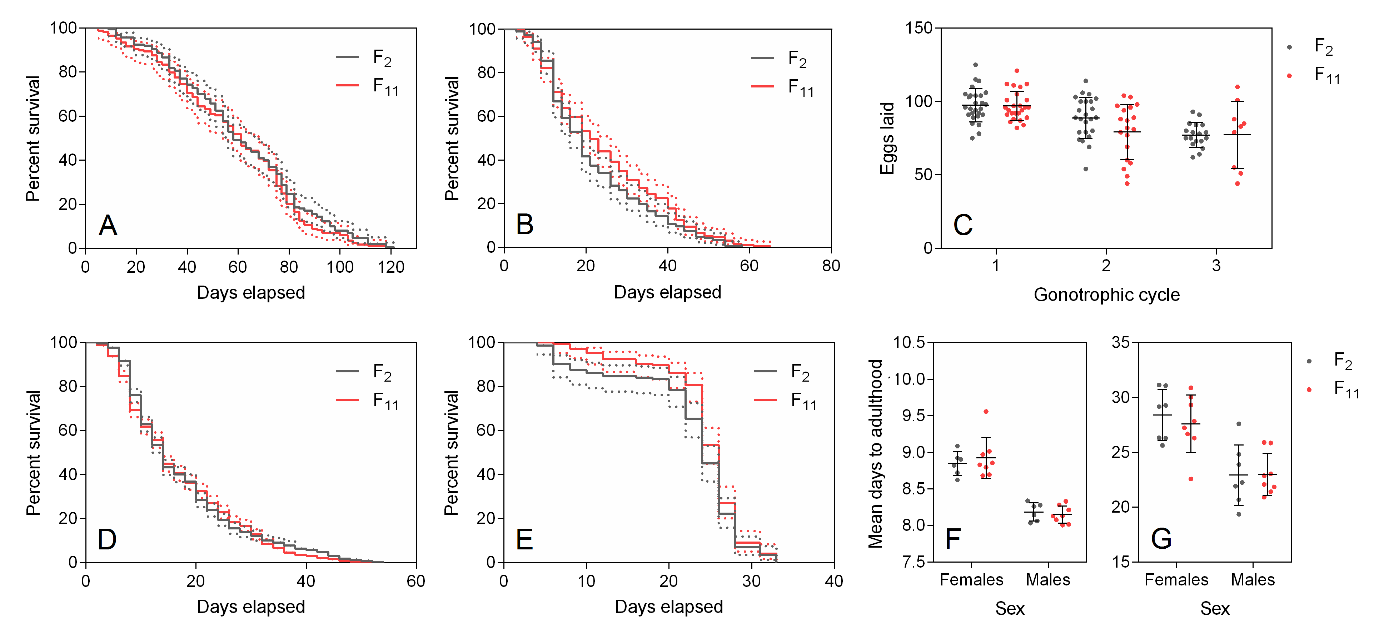


**Figure 1.** Fitness comparisons between Townsville F_2_ (gray) and Cairns F_11_ (red) populations in the laboratory. We scored the longevity of (A) female and (B) male adults, (C) the fecundity of females over three gonotrophic cycles, the survival larvae under when held in (D) groups of 50 or (E) in isolation under starvation conditions and the development time of females and males under (F) high nutrition (1 mg per larva every 2 days) or (G) low nutrition (0.1 mg per larva every 2 days) conditions. Dotted lines on survival graphs represent 95% confidence intervals, while error bars on the fecundity and development time graphs are standard deviations.

*Literature cited*

Axford, J. K., P. A. Ross, H. L. Yeap, A. G. Callahan, and A. A. Hoffmann. 2016. Fitness of *w*AlbB *Wolbachia* infection in *Aedes aegypti*: parameter estimates in an outcrossed background and potential for population invasion. *Am J Trop Med Hyg* 94 (3):507-516.

Ross, P. A., N. M. Endersby, and A. A. Hoffmann. 2016. Costs of three *Wolbachia* infections on the survival of *Aedes aegypti* larvae under starvation conditions. *PLoS Negl Trop Dis* 10 (1):e0004320.
