# Supplementary material for: A comprehensive assessment of inbreeding and laboratory adaptation in Aedes aegypti mosquitoes
Source: Evol Appl. 2018 Dec 17;12(3):572–86. doi: 10.1111/eva.12740 (PMC6383739; doi:10.1111/eva.12740)
Supplement: Supplementary file 5 [file EVA-12-572-s005.docx]

**S2 Appendix.** Fitness comparisons between large *Aedes aegypti* populations (census size 400) and inbred lines from Townsville at F_5_.

*Materials and Methods*

We conducted a set of experiments on the Townsville populations at F_5_ to see if there were any differences between replicate populations after a few generations of being maintained separately. We compared the five large populations at F_5_ and five inbred populations that had experienced two consecutive generations of full-sib mating by F_5_ (S2 Table). We also included Cairns (F_14_) and Innisfail (F_2_) populations for comparison; isofemale lines and small populations were not tested at this stage. We measured larval development time, survival to adulthood and fecundity of each population under two levels of nutrition. Four replicate containers with 50 larvae each (except for Innisfail F_2_ and Cairns F_14_ which had 8 replicates) in 200 mL of water were provided with TetraMin *ad libitum* (high nutrition) or with 0.05 mg of TetraMin per larva every 2 days (low nutrition). For the high nutrition treatment, 20-30 females per population were isolated for fecundity measurements. For the low nutrition treatment, all females that emerged from the containers were isolated for fecundity due to low survival rates.

*Results*

Fecundity under high nutrition conditions did not differ significantly between large and inbred populations (one-way ANOVA: F_1,181_ = 0.126, P = 0.723) but replicates of the large populations differed from each other (F_1,87_ = 3.422, P = 0.012, Figure 1). Under low nutrition conditions fecundity was considerably more variable and differed between replicate inbred populations (Kruskal-Wallis: χ^2^ = 10.519, df = 4, P = 0.033) but not between large and inbred populations (Mann-Whitney U: Z = 1.752, P = 0.080). The survival of inbred larvae to adulthood was consistently lower than for the large populations under high nutrition conditions (Mann-Whitney U: Z = 3.665, P < 0.001). Differences were less clear under low nutrition conditions (Z = 1.488, P = 0.136) due to substantial variation between replicate inbred lines (Kruskal-Wallis: χ^2^ = 13.273, df = 4, P = 0.010). Both male (Mann-Whitney U: Z = 3.963, P < 0.001) and female (Z = 4.693, P < 0.001) development times were greatly extended for inbred lines under high nutrition conditions relative to the large populations. There were also significant differences between replicate inbred populations (Kruskal-Wallis: males: χ^2^ = 13.986, df = 4, P = 0.007, females: χ^2^ = 13.700, df = 4, P = 0.008) and large populations for males (χ^2^ = 11.057, df = 4, P = 0.026) but not females (χ^2^ = 8.443, df = 4, P = 0.077). Effects on development time were less clear under low nutrition conditions as the variation between replicate containers was much higher. Overall, survival to adulthood and female fecundity were drastically reduced, development times were greatly extended, and fitness differences between inbred and large populations became less clear under low nutrition conditions (Figure 1). These results demonstrate that substantial fitness costs arise after only two generations of inbreeding, and differences between replicate populations appeared after only a few generations of being maintained separately in the laboratory.


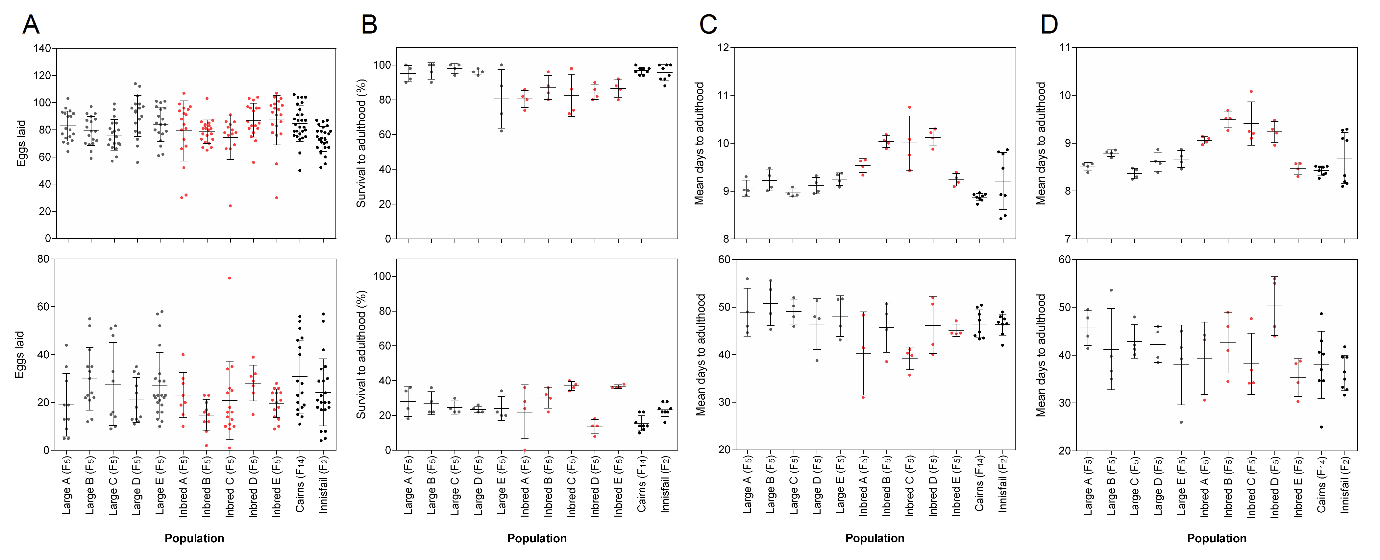


**Figure 1. Fitness comparisons between large populations and inbred lines at F_5_.** Fitness traits were compared between five large populations (gray) and five inbred lines (red) at F_5_ under high nutrition (food provided *ad libitum*, upper panels) and low nutrition (0.05 mg of food per larva every 2 days, lower panels) conditions. Cairns F_14_ and Innisfail F_2_ populations (black) were also included for comparison. Populations were scored for their (A) fecundity, (B) survival to adulthood and development time for (C) females and (D) males. Error bars indicate means and standard deviations.
