# Supplementary material for: A comprehensive assessment of inbreeding and laboratory adaptation in Aedes aegypti mosquitoes
Source: Evol Appl. 2018 Dec 17;12(3):572–86. doi: 10.1111/eva.12740 (PMC6383739; doi:10.1111/eva.12740)
Supplement: Supplementary file 6 [file EVA-12-572-s006.docx]

**S3 Appendix.** The extent of laboratory adaptation in *Aedes aegypti* and comparisons with other insects.


*Methods*

We wanted to see if there was evidence for consistent directional changes in *Aedes aegypti* life history traits resulting from laboratory maintenance, and to assess the magnitude of these changes for comparisons with other studies. We took trait means from each large Townsville population at F_13_ (census size 400) as these were the populations least affected by inbreeding, and compared them to the trait mean of the ancestral population (Townsville F_4/5_) (Figure 1). Traits were compared in terms of proportional changes in trait means per generation (Figure 1A) and standard deviations from the trait mean per generation (Figure 1B), a measure which considers the variation between measurements and not just differences in trait means. When the 95% confidence intervals of the proportional changes did not intersect with zero, we considered these changes to be significant. However, significant changes only indicate a trend in trait means relative to the ancestral population and do not consider the variation within populations.

*Results*

Most traits did not differ from the ancestral population at F_4/5_ and the laboratory populations at F_13_, but other traits exhibited consistent positive or negative shifts in trait means (Figure 1A). Under high nutrition conditions, laboratory populations developed around 0.3 days (3.5%) faster, and wing lengths were 0.08 mm (3%) smaller; though these changes were consistent they reflect only minor shifts. Survival to adulthood in the laboratory populations was also consistently lower under high nutrition conditions and consistently higher under low nutrition conditions, but these differences represent no greater than a few percentage points. When the laboratory and field populations from Cairns and Innisfail were included, fewer traits exhibited consistent changes in one direction (Figure 2); patterns of trait changes with laboratory maintenance were therefore not always consistent.

We compared the extent of adaptation across all traits in this study to data from a review of laboratory adaptation in insects (Hoffmann and Ross 2018). The median proportional change in trait means (unsigned) in our *Ae. aegypti* laboratory populations was 2.54% (n = 86 trait measurements), which was lower than the magnitude of adaptation across all mosquitoes (median = 5.70%, n = 43, Mann-Whitney U: Z = 2.665, P = 0.008) and substantially lower than magnitudes of adaptation across all insects (median = 12.05%, n = 369, Z = 8.841, P < 0.001). Median proportional changes per generation were 0.29% for *Ae. aegypti* in our study (n = 86) and 0.64% across insects in other studies (n = 296, Z = 4.795, P < 0.001). This indicates that mosquitoes, particularly *Ae. aegypti*, undergo less adaptation in response to laboratory rearing conditions than other insects. This is unsurprising given that *Ae. aegypti* are already somewhat adapted to artificial environments, where they preferentially feed on humans and breed in household-linked containers.

**
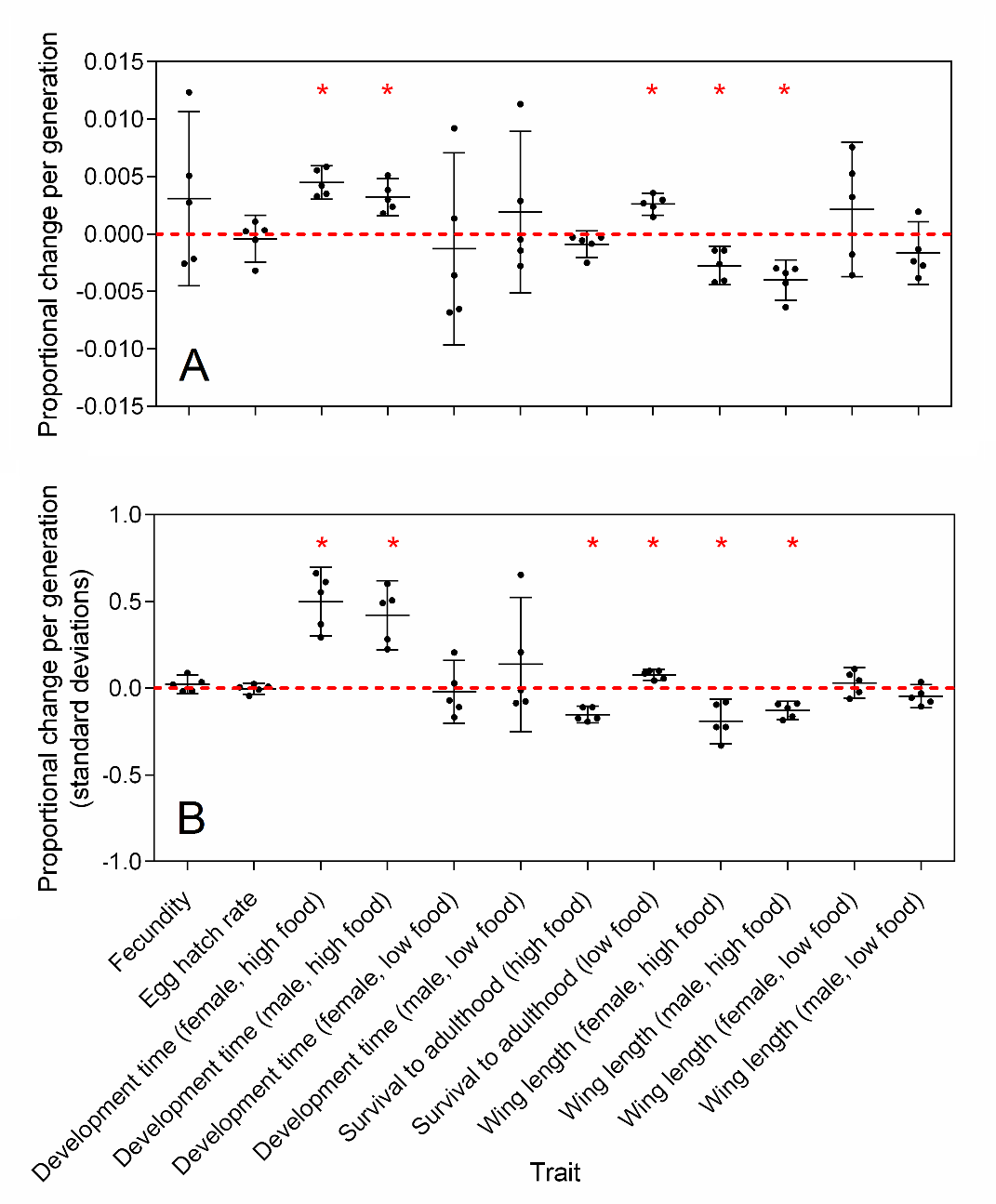
**

**Figure 1. Proportional changes in life history traits in the large populations at F_13_ relative to the ancestral population (Townsville F_4/5_).** Proportional changes are expressed in terms of (A) changes in trait means per generation and (B) standard deviations of trait means per generation. Each data point represents the proportional change in a trait of a single replicate population relative to the ancestral population. The directions of trait changes were adjusted so that positive values indicate increased fitness with laboratory maintenance. Error bars are 95% confidence intervals; red asterisks indicate traits where the 95% confidence intervals do not include zero. Significant changes indicate a trend in trait means relative to the ancestral population but do not consider the variation within populations.


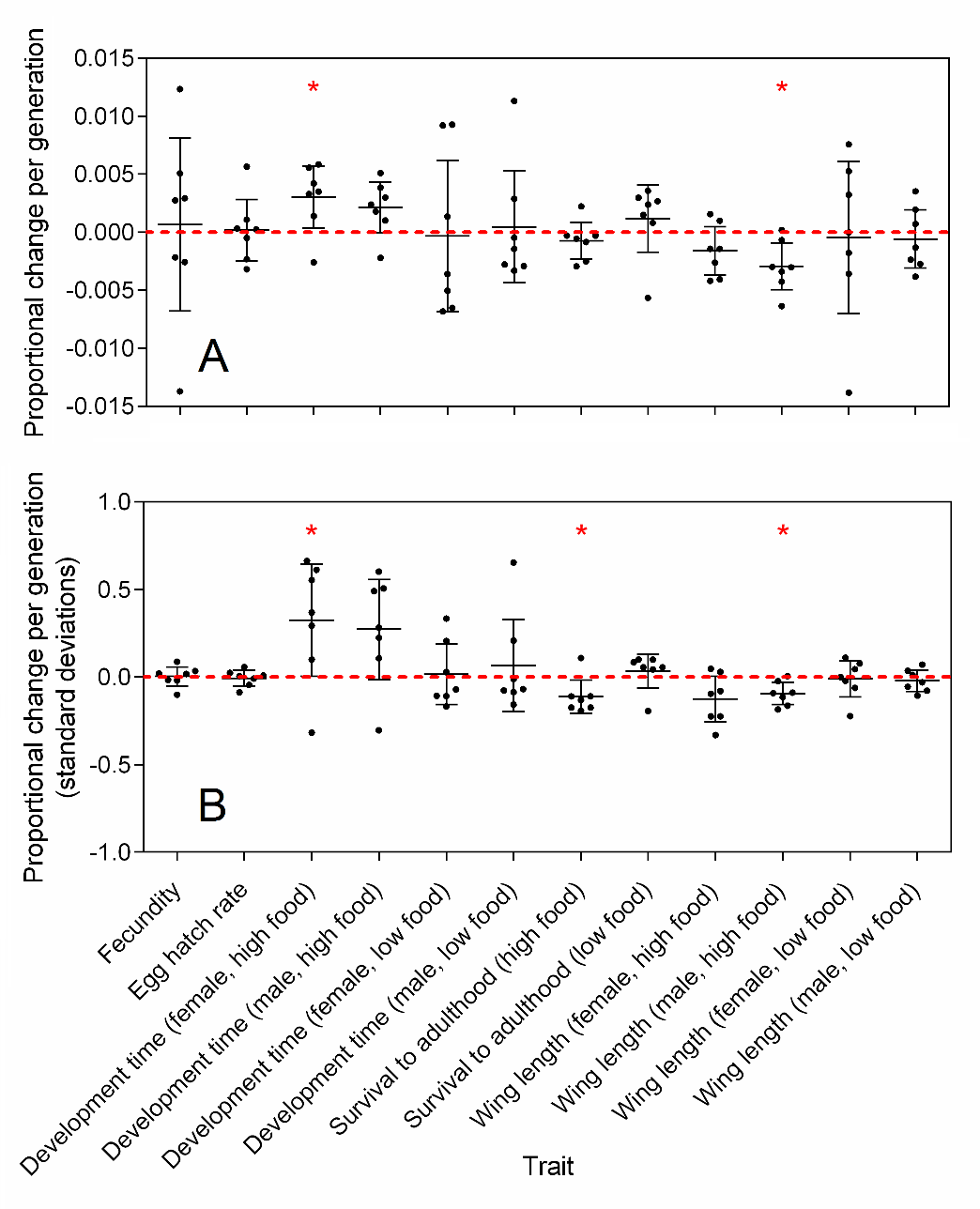


**Figure 2. Proportional changes in life history traits in laboratory populations from Townsville, Cairns and Innisfail relative to their respective ancestral or field populations.** Proportional changes are expressed in terms of (A) changes in trait means per generation and (B) standard deviations of trait means per generation. Each data point represents the proportional change in a trait of a single replicate population relative to the ancestral population. The directions of trait changes were adjusted so that positive values indicate increased fitness with laboratory maintenance. Error bars are 95% confidence intervals; red asterisks indicate traits where the 95% confidence intervals do not include zero. Significant changes indicate a trend in trait means relative to the ancestral or field population but do not consider the variation within populations.

*Literature cited*

Hoffmann, A. A. and P. A. Ross. 2018. Rates and patterns of laboratory adaptation in (mostly) insects. *J Econ Entomol*.
