# Supplementary material for: A comprehensive assessment of inbreeding and laboratory adaptation in Aedes aegypti mosquitoes
Source: Evol Appl. 2018 Dec 17;12(3):572–86. doi: 10.1111/eva.12740 (PMC6383739; doi:10.1111/eva.12740)
Supplement: Supplementary file 7 [file EVA-12-572-s007.docx]

**S4 Appendix.** Outcrossing of an inbred *Aedes aegypti* population. *Methods*

Egg hatch rates for the inbred line (Inbred A) in the mating competitiveness experiment were reduced markedly compared to other populations (Figure 6 main text). To confirm that this result was due to reduced mating competitiveness of inbred males and not reduced fertility of F_2_ females that mated with inbred males, we performed reciprocal crosses between inbred and Cairns F_2_ lines and scored their fecundity and the proportion of eggs that hatched. We then tested offspring from the F_2_ and inbred lines and reciprocal crosses for their development time, fecundity and egg hatch rate to determine the extent to which the fitness of the inbred line was restored when crossed to an outbred population. Eight replicate trays with 50 larvae in 500 mL of water were reared for each cross and provided with TetraMin *ad libitum*. Twenty-five females from each cross were isolated for fecundity and egg hatch proportion measurements for experiments across both generations.

*Results*

We performed reciprocal crosses between Cairns F_2_ and inbred (Inbred A F_18_) populations to assess the effects of each male type on female fecundity (Figure 1A) and egg hatch proportion (Figure 1B). Cairns F_2_ females laid more eggs than inbred females (one-way ANOVA: F_1,97_ = 15.921, P < 0.001) and their fecundity was unaffected by male type (F_1,97_ = 0.370, P = 0.544). Similarly, inbred females had greatly reduced egg hatch proportions compared to Cairns F_2_ females (Mann-Whitney U: Z = 3.182, P = 0.001) regardless of male type (Z = 0.109, P = 0.912). We wanted to see if the fitness of the inbred line could be improved through reciprocal crosses to the Cairns F_2_ population. Larvae that hatched from each cross were scored for their development time (Figure 1C-D). Inbred larvae developed substantially slower than Cairns F_2_ larvae for both females (one-way ANOVA: F_1,14_ = 132.316, P < 0.001) and males (F_1,14_ = 246.030, P < 0.001). Hybrids had greatly improved development times relative to inbred larvae (females: F_1,20_ = 161.238, P < 0.001, males: F_1,20_ = 107.455, P < 0.001) but differed in the extent to which they improved, with the progeny of Cairns F_2_ ♀ × Inbred ♂ developing faster than the progeny of Inbred ♀ × Cairns F_2_ ♂ for both females (F_1,20_ = 9.366, P = 0.010, Figure 1C) and males (F_1,20_ = 16.455, P = 0.002, Figure 1D). Conversely, egg hatch proportions improved to similar extents for the two hybrids, and did not differ significantly from Cairns F_3_ (Kruskal-Wallis: χ^2^ = 2.416, df = 2, P = 0.299, Figure 1F). Fecundity also did not differ between the hybrids and Cairns F_3_ (one-way ANOVA: F_2,58_ = 1.278, P = 0.286) but the fecundity of the inbred population was lower than all other crosses (F_1,81_ = 29.966, P < 0.001, Figure 1E). Overall, the fitness of an inbred line was greatly improved through one generation of outcrossing, though the extent of improvement, at least in terms of development time, depends on which sex is chosen from each group.


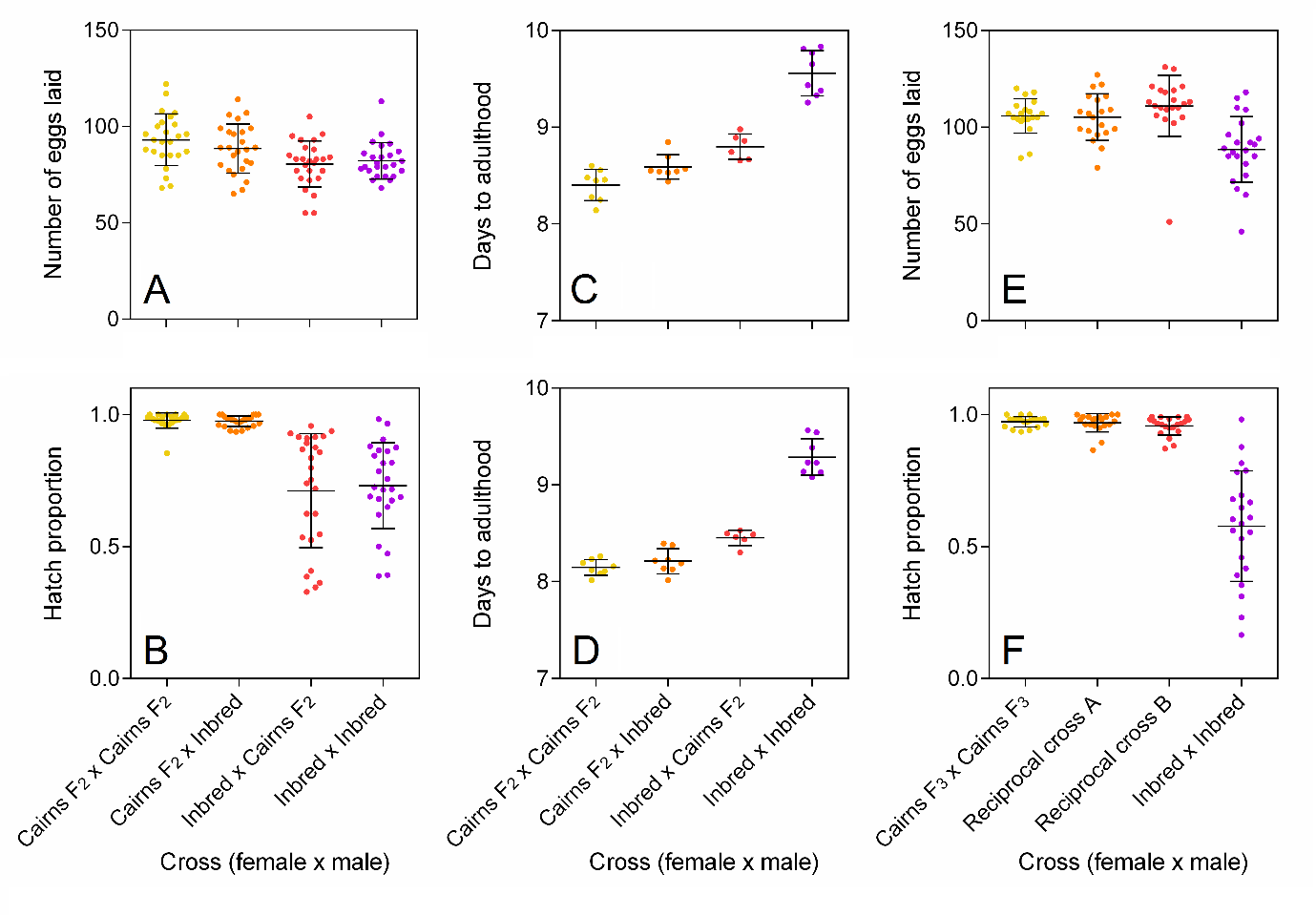


**Figure 1.** (A) Fecundity and (B) egg hatch proportions in crosses between Cairns F_2_ and inbred populations. The resulting progeny were then tested for the development time of (C) females and (D) males, (E) female fecundity and (F) egg hatch proportion. Reciprocal cross A refers to a cross between the male and female progeny of Cairns F_2_ ♀ × Inbred ♂ and reciprocal cross B refers to a cross between the male and female progeny of Inbred ♀ × Cairns F_2_ ♂. Error bars are standard deviations.
