# Supplementary material for: A comprehensive assessment of inbreeding and laboratory adaptation in Aedes aegypti mosquitoes
Source: Evol Appl. 2018 Dec 17;12(3):572–86. doi: 10.1111/eva.12740 (PMC6383739; doi:10.1111/eva.12740)
Supplement: Supplementary file 8 [file EVA-12-572-s008.docx]

**S5 Appendix.** Larval competitiveness of *Aedes aegypti* laboratory populations.

*Materials and Methods*

The Cairns populations at F_2_, F_7_ or F_27_ and the inbred line (Inbred A) at F_18_ were tested for larval competitiveness. Fifty 1^st^ instar larvae from each population and 50 *w*AlbB-infected larvae were added to containers with 500 mL of water, with eight replicate containers each. *w*AlbB-infected larvae were used as a standard competitor, as they can be distinguished from the other populations by qPCR (Lee et al. 2012; Axford et al. 2016). Containers were provided with 0.04 mg of TetraMin per larva every two days; this nutrition regime was chosen to reflect highly competitive conditions and result in low survival rates. All adults that emerged were stored in ethanol and screened for their *Wolbachia* infection status using previously described methods (Lee et al. 2012; Axford et al. 2016). The frequency of *Wolbachia* infection in the emerging adults was used to determine the relative competitiveness of each population; higher frequencies of adults without *Wolbachia* indicate increased competitiveness of the experimental population.

*Results*

Approximately 18.5% of larvae in the experiment survived to adulthood and development times were greatly extended relative to other experiments under high nutrition conditions (Figure 1, c.f. Figure 2 in main text). All adults that emerged were screened for their *Wolbachia* infection status, with the proportion from each population that were uninfected providing an estimate of their larval competitiveness (Figure 1A). No differences in larval competitiveness were found between the Cairns laboratory populations (Kruskal-Wallis: χ^2^ = 1.915, df = 2, P = 0.384). The proportion of uninfected adults for the inbred line was substantially reduced compared to the other populations, indicating greatly reduced larval competitiveness (Mann-Whitney U: Z = 4.156, P < 0.001). Development times of experimental larvae were slower than larvae from the standard competitor for females (Mann-Whitney U: Z = 3.522, P < 0.001, Figure 1B) but not for males (Z = 0.934, P = 0.352, Figure 1C).

*
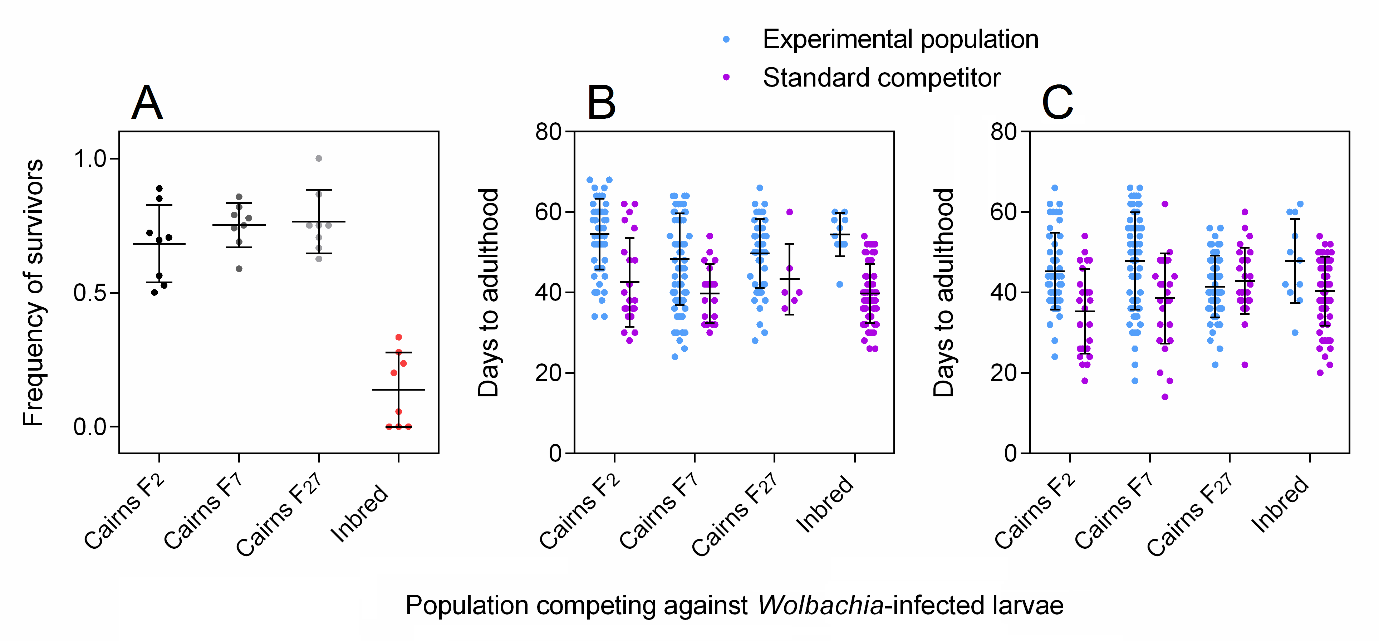
*

**Figure 1.** Relative larval competitive ability of the Cairns F_2_, F_7_, F_27_ and inbred F_18_ (Inbred A) populations *versus* *Wolbachia*-infected larvae when added in equal proportions at the start of the experiment. (A) The proportion of larvae surviving to adulthood that were not infected with *Wolbachia*. Higher frequencies indicate a greater competitive ability of the experimental population relative to the *Wolbachia*-infected standard competitor. Frequencies were calculated from all adults that emerged from each container, ranging from 10 to 29 adults per data point (males and females were combined). (B-C) Development times for individual (B) females and (C) males that survived to adulthood. Development time data were separated into groups for experimental individuals (blue) and *Wolbachia*-infected individuals (purple). Each data point for (B) and (C) represents a single individual rather than the mean development time for an entire container. Error bars are standard deviations.

*Literature cited*

Axford, J. K., P. A. Ross, H. L. Yeap, A. G. Callahan, and A. A. Hoffmann. 2016. Fitness of *w*AlbB *Wolbachia* infection in *Aedes aegypti*: parameter estimates in an outcrossed background and potential for population invasion. *Am J Trop Med Hyg* 94 (3):507-516.

Lee, S. F., V. L. White, A. R. Weeks, A. A. Hoffmann, and N. M. Endersby. 2012. High-throughput PCR assays to monitor *Wolbachia* infection in the dengue mosquito (*Aedes aegypti*) and *Drosophila simulans*. *Appl Environ Microbiol* 78 (13):4740-4743.
