# Supplementary material for: A comprehensive assessment of inbreeding and laboratory adaptation in Aedes aegypti mosquitoes
Source: Evol Appl. 2018 Dec 17;12(3):572–86. doi: 10.1111/eva.12740 (PMC6383739; doi:10.1111/eva.12740)
Supplement: Supplementary file 9 [file EVA-12-572-s009.docx]

**S6 Appendix.** Adult body size of *Aedes aegypti* laboratory populations.

We measured wing length from a random subset of adults emerging from the larval development experiment to estimate the body size of each population under different nutritional conditions (Ross et al. 2014). Wing lengths under high nutrition conditions were much larger than under low nutrition conditions (one-way ANOVA: F_1,918_ = 221.749, P < 0.001) and differences between populations were more distinct. Under high nutrition conditions there was a clear cost of inbreeding to wing length; adults from inbred lines were much smaller than adults from large populations (females: F_1,82_ = 67.189, P < 0.001, males: F_1,82_ = 22.804, P < 0.001). There were substantial differences in wing length between replicate isofemale lines (females: F_4,45_ = 8.303, P < 0.001, males: F_4,45_ = 10.751, P < 0.001) and inbred lines (females: F_3,30_ = 20.703, P < 0.001, males: F_3,30_ = 9.729, P < 0.001) and smaller, but still significant, differences between females of the large (F_4,45_ = 3.102, P = 0.024) and small (F_4,44_ = 2.777, P = 0.038) populations. Wing lengths of adults from the large populations at F_13_ were smaller than those from the ancestral population at F_4/5_ (females: F_1,58_ = 10.472, P = 0.002, males: F_1,58_ = 10.519, P = 0.002) which could reflect adaptation to artificial rearing conditions. However, there were no differences between the laboratory and field populations from Cairns and Innisfail (all P > 0.05).

**
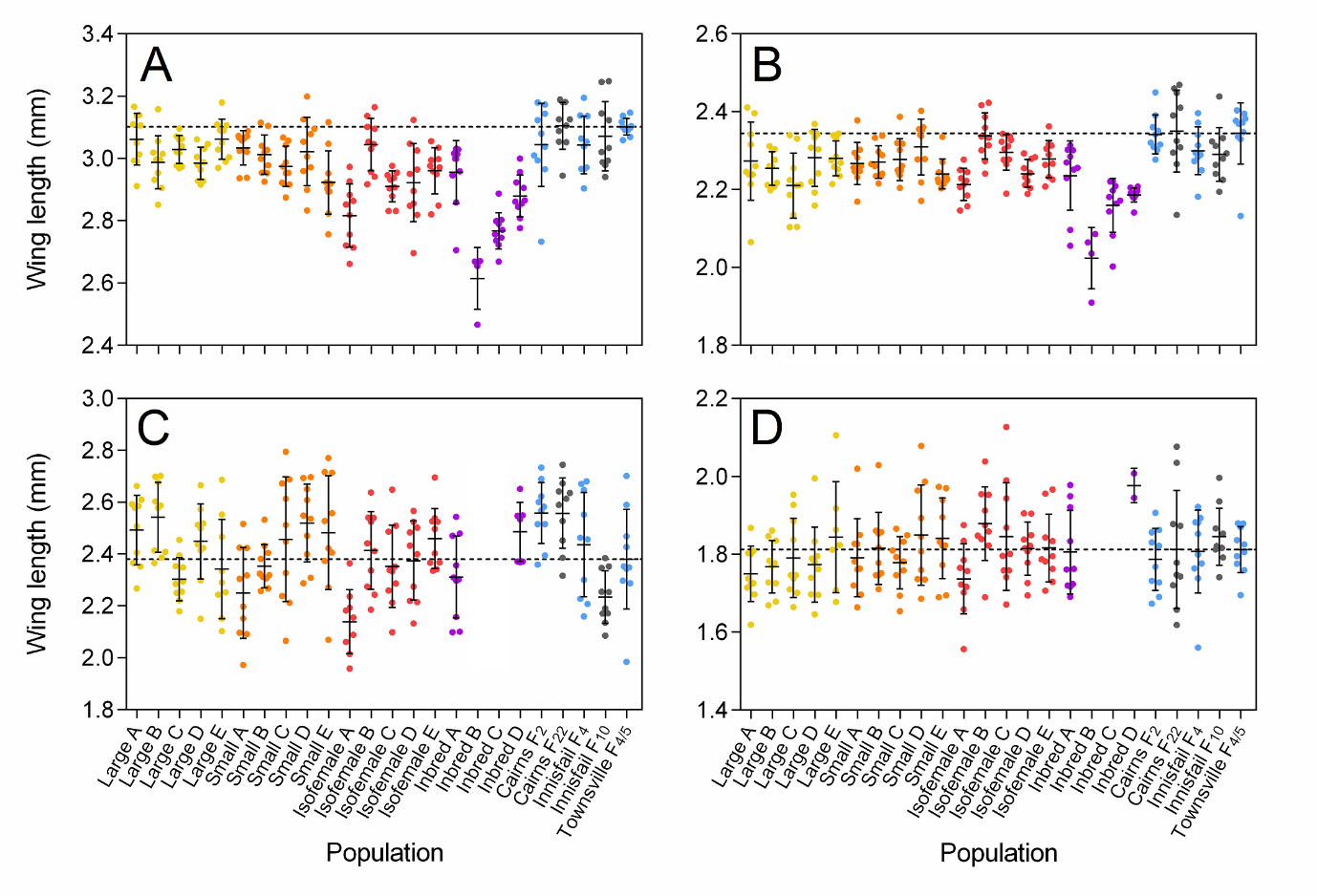
**

**Figure 1.** Wing length of *Aedes aegypti* F_13_ laboratory populations maintained at different census sizes. Wing lengths were measured from (A&C) female and (B&D) male adults when reared under (A&B) high nutrition (food provided *ad libitum*) and (C&D) low nutrition (0.1 mg of TetraMin^®^ per larva every 2 days) conditions. Replicates of the large populations (Townsville F_13_, census size 400) are in yellow, small populations (Townsville F_13_, census size 100) in orange, isofemale lines (Townsville F_13_) in red and inbred lines (Townsville F_13_) in purple. Other laboratory populations are shown in gray and ancestral / field populations in pale blue. Up to ten wings were measured for each group, though some inbred lines had less than 10 adults available, and several measurements were discarded due to damaged wings. Inbred lines B and C were not tested under low nutrition conditions. The dashed line represents the mean wing length of the Townsville F_4/5_ ancestral population. Error bars are standard deviations.
